# Jun N-Terminal Kinase Inhibitor Suppresses CASK Deficiency-Induced Cerebellar Granular Cell Death in MICPCH Syndrome Model Mice

**DOI:** 10.3390/cells14100750

**Published:** 2025-05-20

**Authors:** Qi Guo, Emi Kouyama-Suzuki, Yoshinori Shirai, Katsuhiko Tabuchi

**Affiliations:** 1 Department of Molecular and Cellular Physiology, Shinshu University School of Medicine, Matsumoto 390-8621, Japan; gq_2222@126.com (Q.G.); emi_suzuki@shinshu-u.ac.jp (E.K.-S.); yoshirai@shinshu-u.ac.jp (Y.S.); 2 Department of NeuroHealth Innovation, Institute for Biomedical Sciences, Interdisciplinary Cluster for Cutting Edge Research, Shinshu University, Matsumoto 390-8621, Japan

**Keywords:** CASK, cerebellar granule cell, reactive oxygen species, MAPK, JNK inhibitors, MICPCH syndrome

## Abstract

Microcephaly with pontine and cerebellar hypoplasia (MICPCH) syndrome is a severe neurodevelopmental disorder caused by a deficiency in the X-linked gene calcium/calmodulin-dependent serine protein kinase (CASK). A better understanding of the role of CASK in the pathophysiology of neurodevelopmental disorders may provide insights into novel therapeutic and diagnostic strategies for MICPCH syndrome and other neurodegenerative diseases. To investigate this, we generated CASK knockout (KO) cerebellar granule (CG) cell culture from CASK floxed (CASK^flox/flox^) mice by infecting lentiviruses expressing codon-improved Cre recombinase (iCre). We performed RNA-sequencing (RNA-seq) on these cells and found that CASK-KO CG cells underwent apoptosis by activating intracellular Jun N-terminal kinase (JNK) signaling and upregulating reactive oxygen species (ROS)-related gene expression. We also performed mouse gait analysis and limb clasping behavior experiments on trans-heterozygous CASK-KO and Hprt-eGFP (CASK^+/-^ Hprt^eGFP/+^) mice. The CASK^+/-^ Hprt^eGFP/+^ mice exhibited cerebellar ataxic phenotypes as judged by the scores of these experiments compared to the CASK wild-type control (CASK^+/+^ Hprt^eGFP/+^) mice. Interestingly, the administration of the JNK inhibitor, JNK-IN-8, in CASK-KO CG cell cultures increased CG cell survival by reducing ROS generation. Moreover, injection of JNK-IN-8 into the cerebellum of CASK^+/-^ Hprt^eGFP/+^ mice suppressed CG cell death and alleviated cerebellar ataxic phenotypes in vivo. In conclusion, JNK-IN-8 suppresses the cell death and activation of the ROS pathway in CASK-KO CG cells in both in vitro and in vivo models, suggesting its potential as a therapeutic strategy for cerebellar neurodegeneration in MICPCH syndrome.

## 1. Introduction

Microcephaly with pontine and cerebellar hypoplasia (MICPCH) syndrome is a severe neurodevelopmental disorder with numerous symptoms associated with microcephaly and pontocerebellar hypoplasia, usually found in girls, and lethality in boys [1,2,3]. MICPCH syndrome is caused by the deficiency of the X-linked gene calcium/calmodulin-dependent serine protein kinase (CASK) that encodes a membrane-associated guanylate kinase (MAGUK) family protein, containing a CaMK domain, two L27 domains, a PDZ domain, an SH3 domain, and a C-terminal guanylate kinase domain [4]. CASK has been originally identified as an intracellular binding partner for Neurexins [5] and considered to function in regulating the formation and maintenance of synapses and neurotransmitter release in the brain [6]. CASK knockout (KO) mice showed significant developmental abnormalities and lethality [7]. The excitatory/inhibitory (E/I) balance is disrupted in cultured neurons derived from CASK-KO mice. Female CASK heterozygote KO (CASK-hKO) mice may be the models that mimic the genetic background of most MICPCH syndrome. These mice are subjected to the effects of X-chromosome inactivation, resulting in a mosaic distribution of CASK-expressing and CASK-KO cells in the cerebral cortex [8]. Patch-clamp electrophysiology in acute brain slices has shown that excitatory synaptic input to CASK-KO pyramidal neurons is increased, while inhibitory synaptic input is reduced in CASK-hKO mice [8]. This is attributed to insufficient transcription of Grin2B, which encodes the GluN2B subunit of the N-methyl-D-aspartate (NMDA) receptor, due to a deficiency in the complex formed between the guanylate kinase domain of CASK and the T-box transcription regulatory factor TBR1 [8].

Mutations in CASK identified in human patients have been shown to affect brain development and function. In vitro experiments demonstrated that these mutations cause neurodegeneration and apoptosis in cerebellar granule cells (CG cells) [9]. However, the detailed interaction between the CASK deficiency and the degeneration of CG cells has not been completely investigated. A better understanding of the role of CASK in the maintenance of the cerebellar structure may provide insights into the prevention of cerebellar hypoplasia in MICPCH syndrome.

Ding et al. reported that the knockdown of CASK in hepatocellular carcinoma (HCC) activates the Jun N-terminal kinase (JNK), a kinase that belongs to the family of stress-activated serine/threonine protein kinases of the mitogen-activated protein kinase (MAPK) pathway [10]. JNK signaling plays a key role in cell death by regulating reactive oxygen species (ROS) [11,12], and the administration of the JNK inhibitor significantly attenuates CASK deficiency-mediated autophagic cell death [10]. This finding suggests that CG cell death in CASK-KO mice may also be involved in the JNK and MAPK pathways, and inhibiting such pathways may rescue CG cell survival.

To investigate the molecular mechanism underlying cerebellar neurodegeneration caused by CASK deficiency, we performed RNA-sequencing (RNA-seq) analysis on CASK-KO CG cell culture. We identified that the JNK pathway-related genes were upregulated in these cells. The administration of JNK inhibitor rescued the CASK-KO CG cells in both in vitro and in vivo. These results suggest that JNK inhibitors have the potential to serve as a therapeutic strategy for cerebellar neurodegeneration in MICPCH syndrome.

## 2. Materials/Subjects and Methods

### 2.1. Animals and Housing Conditions

Floxed CASK (CASK^flox/flox^) mice and wild type mice (CASK^+/+^)(B6;129-Casktm1Sud/J, JAX Stock #006382) were as described previously [9], Hprt-eGFP mice (B6;129-Hprt<tm1(CAG-eGFP-NLS)Koba>, RBRC09532) were obtained from RIKEN BioResource Research Center. Male homozygous CASK-KO (CASK^-/Y^) mice and female heterozygous CAS-KO mice (CASK^+/-^ mice) were obtained by crossing CASK^flox/flox^ mice and ZP3-Cre mice [13] (C57BL/6-Tg (Zp3-cre)93Knw/J, JAX Stock #003651). Heterozygous CASK-KO with Hprt-eGFP (CASK^+/-^ Hprt^eGFP/+^) mice were obtained by crossing female CASK^+/-^ mice and Hprt-eGFP mice.

All animals were maintained with a 12:12 light-dark cycle with food and water ad libitum. The room temperature was maintained at 23 ± 2 °C. Animal experiments were reviewed by the Committee for Animal Experiments and were approved by the president of Shinshu University (approved on 17 March 2022, approved number, 021045). The methods were carried out in accordance with the Regulations for Animal Experimentation of Shinshu University and the ARRIVE guidelines 2.0.

### 2.2. Plasmid and Lentivirus Preparation

Lentivirus-CTL: EGFP was removed by digestion with BamHI and EcoRI from pFSy(1.1)GW (Addgene #27232) and self-ligated. pFSyGW-iCre (lentivirus-iCre): BamHI-iCre-EcoRI was cloned into the BamHI/EcoRI site of pFSy(1.1)GW. Empty lentivirus (pFSy-control, lentivirus-CTL) was used as a control. Construction of all other plasmids used in this study was described previously [9].

### 2.3. Cell Culture

Primary CG cells were prepared from wild-type C57BL/6, CASK^floxed/floxed^, or CASK^+/flox^Hprt^eGFP/+^ mice at P6, as described previously [9,14]. In short, isolated cerebella were incubated with phosphate-buffered saline (PBS) containing 1% trypsin (Sigma-Aldrich, St. Louis, MO, USA) and 0.1% DNase I (Sigma-Aldrich) for 3 min and dissociated by passing through a fire-polished Pasteur pipette in PBS containing 0.05% DNase I, 0.03% trypsin inhibitor (Sigma-Aldrich), and 2 mM MgCl_2_.Cells were plated on coverslips coated with mouse laminin (ThermoFisher Scientific, Waltham, MA, USA) and poly-L-lysine (Sigma-Aldrich) at a density of 2 × 10^6^ cells/well, cultured in Neurobasal-A (ThermoFisher Scientific) supplemented with 2% B-27 supplement (ThermoFisher Scientific), 5% fetal bovine serum (FBS) (Biowest, Nuaillé, France), 100 U/mL of Antibiotic-Antimycotic Mixed (Nacalai tesque, Kyoto, Japan), and 2 mM GlutaMAX I (ThermoFisher Scientific) for 24 h at 37 °C in a 5% CO_2_ atmosphere, then cultured in the same medium without FBS.

The cultured cells were infected with either an empty lentivirus (pFSy(1.1)GW) or the lentivirus expressing codon-improved Cre recombinase (iCre) (pFSyGW iCre) at DIV1. After the first infection, the culture medium was replaced with fresh medium, followed by infection with an empty lentivirus or a lentivirus expressing CASK.

For drug effect testing, primary CG cells prepared from wild-type C57BL/6, CASK^floxed/floxed^, or CASK^+/flox^Hprt^eGFP/+^ mice, infected with lentiviruses expressing iCre, were divided into drug-treated and control groups. Three hours after the initial lentiviral infections, the culture medium of the drug-treated group was replaced with fresh medium containing JNK inhibitors at final concentrations of 0.1 μM, 1 μM, 10 μM, 20 μM, 50 μM, or 100 μM (see Table S2). The control group received an equivalent volume of DMSO (0.1%).

Cells were harvested at DIV4 for RNA extraction and fixed at DIV7 with 4% paraformaldehyde (PFA) and 4% sucrose for subsequent experiments. For each independent experiment, cerebella from 4 to 5 mice were used. In total, approximately 50 CASK^+/+^ mice, 50 CASK^flox/flox^ mice, and 12 CASK^+/flox^Hprt^eGFP/+^ mice were used for cerebellar granule cell cultures.

### 2.4. RNA Extraction and Purification

Total RNA was extracted using TRIzol (Ambion, Carlsbad, CA, USA), followed by the manufacturer’s instructions, and purified with the PureLink™ RNA Mini Kit (ThermoFisher Scientific, Waltham, MA, USA). After purification, the total RNA was stored at −80 °C for further study.

### 2.5. RNA-Sequencing Analysis

RNA-seq was conducted by Takara Bio (TAKARA BIO INC., Shiga, Japan). Briefly, the reverse transcription was performed with the SMART (Switching Mechanism at 5’ End of RNA Template) method with the SMART-Seq Stranded Kit. Then, the PCR product purification was conducted using AMpure XP (Beckman Coulter, Brea, CA, USA). The RNA-seq library was prepared by the Nextera XT DNA Library Prep Kit. Sequence was performed with NovaSeq 6000 (Illumina, San Diego, CA, USA). The results were normalized by using the TPM (Transcripts Per Million), data visualization was performed using RStudio [15] (R 4.3.0; Posit Software; Boston, MA, USA), gene ontology analysis was performed with the Metascape [16] and gene set enrichment analysis (GSEA) [17,18]. Three biological replicates of each group were prepared to ensure statistical significance.

### 2.6. Stereotaxic Surgery

P6 CASK^+/-^ Hprt^eGFP/+^ or CASK^+/+^ Hprt^eGFP/+^ mice were anesthetized on ice. Using Neuros syringes (65460-02, Hamilton, USA), approximately 10 mg/mL (2% DMSO, 30% PEG 300, 5% Tween 80, 63% ddH_2_O) JNK-IN-8 or DMSO, 6.6 mg/kg was injected bilaterally in the following stereotactic coordinates of the cerebellum–anterioposterior (AP)—1.2 mm, mediolateral (ML) ± 1.0 mm, and dorsoventral (DV)—1.3 mm, taking lambda as the reference point using a stereotaxic device (Narishige, Tokyo, Japan). In total, 2 μL of drug and 1 μL of 0.4% trypan blue were injected into each mouse. After the surgery, keep the heater on for 30 min before moving back to the mother. The mice were kept for 2 weeks after injection, before further experiments. 24 CASK^+/-^ Hprt^eGFP/+^ mice and 24 CASK^+/+^ Hprt^eGFP/+^ mice were used for Stereotaxic Surgery. 10 CASK^+/-^ Hprt^eGFP/+^ mice, 10 CASK^+/+^ Hprt^eGFP/+^ mice were used for bodyweight assessment (*n* = 5 in each group).

### 2.7. Limb-Clasping Scoring

One week before scoring, mice were allowed to acclimate to the vivarium. Mice were held by the tail for 60 s and recorded by the GoPro 9 (GoPRO, San Mateo, CA, USA). During tail suspension, limb-clasping behavior was evaluated with the following standards: 0—No limb clasping. Normal escape extension: 1—One hind limb exhibits incomplete splay and loss of mobility. Toes exhibit normal splay; 2—Both hind limbs exhibit incomplete splay and loss of mobility. Toes exhibit normal splay; 3—Both hind limbs exhibit clasping with curled toes and immobility; 4—Forelimbs and hind limbs exhibit clasping and are crossed, curled toes and immobility [19]. Seven mice (*n* = 7) were tested in each group.

### 2.8. Mouse Foot Gait Analysis by Ink

Habituate mice to the testing room and gait tunnel with the room light. Place the food at the surface level at the end of the gait tunnel to serve as a goal box. Mice’s forelimbs are coated with red ink, and the hindlimbs are coated with blue ink before they walk across a white paper. The papers with paw prints were used to measure the toe spread, stride length, and stride width [20]. Five mice (*n* = 5) were tested in each group.

### 2.9. Data Visualization and Statistical Analyses

GraphPad (version 10.2.3(403), GraphPad Software; San Diego, CA, USA) and RStudio (R 4.3.0) were used for data visualization, and the quantitative variables are expressed as the mean ± standard error of the mean (SEM). Statistical data analyses were performed using GraphPad (version 10.2.3(403) with two-way analysis of variance (2-way ANOVA) followed by Tukey’s correction, *p* < 0.05 viewed as statistical significance between each group.

### 2.10. Additional Materials and Methods

Additional methods are described in Supplementary Methods. Sequences of primers are listed in Table S1. Reagents and antibodies’ cat numbers are listed in Table S2. Original blot images are shown in Original Data 1. We used ChatGPT 4 for English editing of our manuscript.

## 3. Results

### 3.1. CASK Deletion Causes Activation of JNK Signaling and Apoptosis Pathway in CG Cells

To investigate the molecular mechanism underlying cerebellar hypoplasia in MICPCH syndrome, we first studied postnatal day 0 (P0) CASK^-/Y^ mice. We found the body size of CASK^-/Y^ mice was significantly smaller than that of CASK^+/Y^ mice (Figure S1A). Morphological abnormality was also observed in the H&E-stained cerebellar slices in CASK^-/Y^ mice (Figure S1B). Since CASK^-/Y^ mice die at P0, the capability of in vivo analysis in CASK^-/Y^ mice was limited.

**Figure 1:**
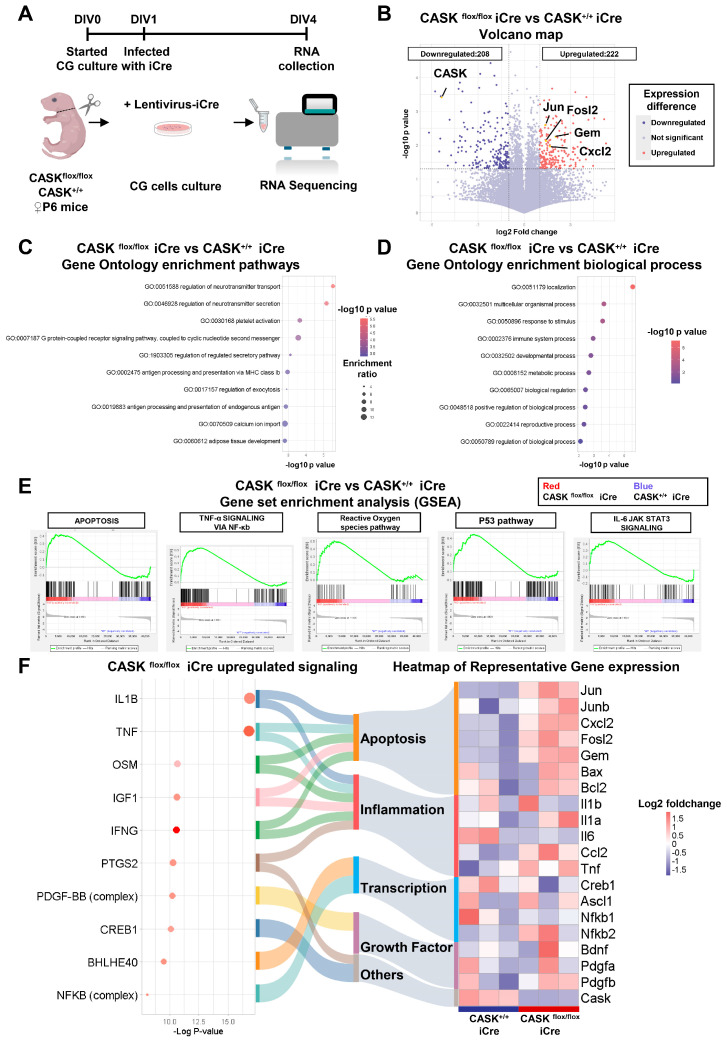
RNA-seq analysis showed activation of JNK signaling and apoptosis pathway in CASK^flox/flox^ iCre CG cells. (**A**). Experimental design of the RNA-seq analysis. P6 CASK^flox/flox^ and CASK^+/+^ mice were used for CG cell culture. Cells were infected with iCre in DIV1, RNA was collected in DIV4, and Protein was collected in DIV5. (*n* = 3 in each group). (**B**). Volcano map of gene expression difference between CASK^flox/flox^ iCre CG cells and CASK^+/+^ iCre CG cells. Red indicated genes upregulated in CASK^flox/flox^ iCre CG cells, blue indicated genes downregulated in CASK^flox/flox^ iCre CG cells. (Threshold: *p* < 0.05, Fold change over2). (**C**). The top 10 Gene Ontology enrichment pathways in CASK^flox/flox^ iCre CG cells analyzed by Metascape. (**D**). The top 10 Gene Ontology enrichment processes in CASK^flox/flox^ iCre CG cells analyzed by Metascape. (**E**). Representative Gene Set Enrichment Analysis (GESA) results of apoptosis-related pathways. (**F**). Upregulated signaling in CASK^flox/flox^ iCre CG cells was analyzed using IPA analysis, and the heatmap of the related gene expression detected by RNA-seq.

We then decided to study gene expression profiling in CASK-deficient cells in comparison with that in wild-type cells. For this, we performed RNA-seq analysis in cultured CG cells. We prepared dissociated CG cell cultures from CASK^flox/flox^ and CASK^+/+^ mice and infected them with lentiviruses expressing iCre at day in vitro (DIV) 1 to generate CASK-KO and wild-type control CG cell cultures (Figure 1A). Since CASK-KO CG cells began to undergo cell death at DIV4, we collected mRNA from CASK-KO and control CG cell cultures at DIV4 for RNA-seq analysis. Compared to the CASK^+/+^ iCre CG cell, 222 genes were significantly upregulated and 208 genes were downregulated (*p* < 0.05; Fold change >2, *n* = 3) in CASK^flox/flox^ iCre CG cells (Figure 1B). The expression of CASK in CASK^flox/flox^ iCre CG cells was significantly (*p* < 0.001) decreased compared to that in CASK^+/+^ iCre CG cell in the RNA-seq results, as expected (Figure 1B). We found the expression levels of JNK signaling and apoptosis-related genes such as c-Jun (*p* < 0.01), Fosl2 (*p* < 0.01), Gem (*p* < 0.01) and Cxcl2 (*p* < 0.05) were significantly increased in CASK^flox/flox^ iCre CG cells compared to those in CASK^+/+^ iCre CG cells (Figure 1B). We selected genes of which expression were significantly changed in CASK-KO CG cells compared to that in wild-type cells and performed Gene Ontology analysis (Figure S2A, 430 in total). The most significantly changed ones were related to the regulation of the neurotransmitter transport pathway (Figure 1C) and the localization process (Figure 1D). This suggests that the deletion of CASK may cause abnormality in neurotransmission, highlighting the significance of CASK in the synaptic function, also in CG cells. The gene ontology analysis also highlighted essential biological processes such as immune system process, metabolic process (Figure 1D). GSEA indicated that cell death related signaling such as apoptosis and Reactive Oxygen Species (ROS) pathway were enhanced in CASK^flox/flox^ iCre CG cells (Figure 1E). In addition, inflammation related pathways such as TNF-α Signaling, P53, and IL-6 JAK STAT3 signaling in CASK^flox/flox^ iCre CG cells were also enhanced (Figure 1E). To further investigate the molecular interaction, we conducted IPA signaling analysis for the representative genes and found that the CASK deletion caused upregulation of the apoptosis and inflammation signaling, such as IL-1β and Tnf, (Figure 1F). These results suggest that the CASK deletion causes the activation of JNK signaling and apoptosis pathway in CG cells.

### 3.2. The Levels of p-c-Jun and p-JNK in CASK^flox/flox^ iCre CG Cells Are Significantly Increased

**Figure 2:**
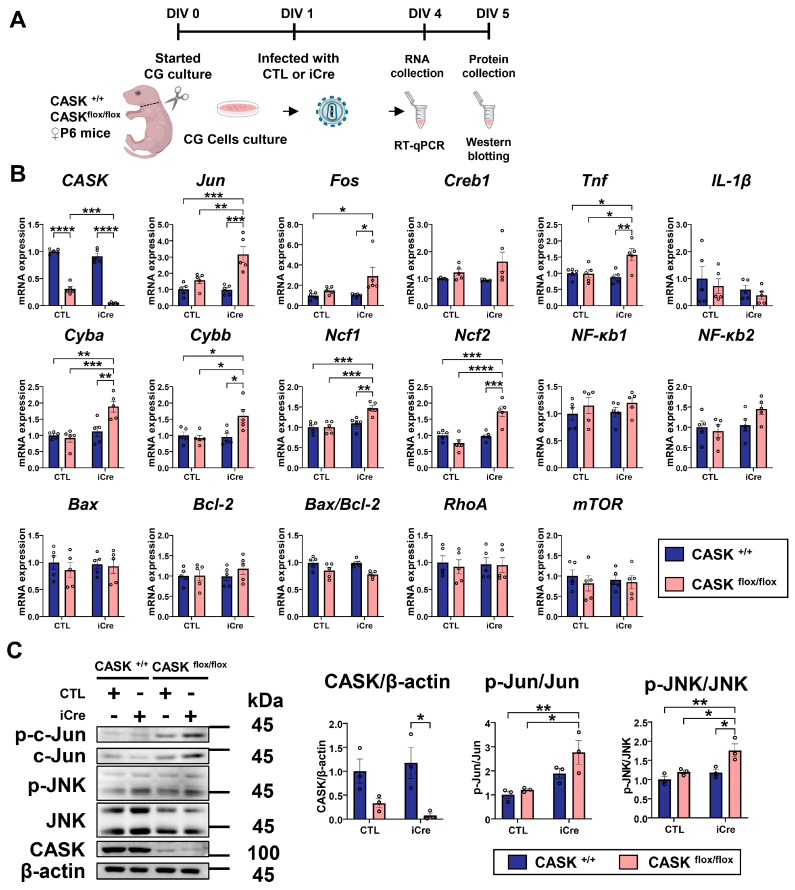
Realtime-qPCR analysis and Western blotting confirmed the activation of JNK signaling in CASK^flox/flox^ iCre CG cells. (**A**). Experimental design of realtime-qPCR and Western blotting. CG cells were infected with lentivirus-iCre and lentivirus-CTL in DIV1, and RNA and protein samples were collected in DIV4. (**B**). Realtime-qPCR analysis on CG cells. (*n* = 5 in each group). (**C**). Western blotting analysis on CG cells. (*n* = 3 in each group) * indicates difference between the groups. * indicates *p* < 0.05; ** indicates *p* < 0.01; *** indicates *p* < 0.001 and **** indicates *p* < 0.0001.

To confirm the RNA-seq results, we performed realtime-qPCR and Western blotting analysis in the extracts from CG cell cultures infected with lentivirus expressing iCre (Figure 2A). The expression of CASK in CASK^flox/flox^ iCre was almost undetectable in these experiments indicating gene removal by Cre recombination was successful (Figure 2B). Of note, the expression level of CASK in CASK^flox/flox^ control (CTL) cells was reduced to approximately 33% of that in CASK^+/+^ cells, as the insertion of loxP sites disrupts normal CASK expression in this hypomorphic allele [7]. Note that the expression of genes related to JNK signaling, such as c-Jun (*p* < 0.001) and c-Fos (*p* < 0.001), in CASK^flox/flox^ iCre CG cells was significantly upregulated compared to the CASK^+/+^ iCre CG cells. The expression of ROS-related genes such as Cyba, Cybb, Ncf1, and Ncf2 was also significantly upregulated in CASK^flox/flox^ iCre-infected CG cells. On the other hand, gene expressions such as IL-1β, NF-κb1, NF-κb2, and Bax/Bcl-2 were not significantly changed between each group (Figure 2B). Therefore, we decided to focus on the protein phosphorylation changes in JNK signaling and ROS pathway.

In Western blotting analysis on these cells, we found the phosphorylated c-Jun (*p* < 0.05) and JNK (*p* = 0.1725) increased in CASK^flox/flox^ iCre CG cells compared to the CASK^+/+^ CTL CG cells (Figure 2C). This suggests the knockout of CASK promotes phosphorylation signaling of JNK, which will cause cell apoptosis in CG cells. These results indicate that the inhibition of JNK signaling may be effective for preventing cell death in CASK-deficient CG cells.

### 3.3. Administration of JNK Inhibitors Promotes the Survival Rate in CASK^flox/flox^ iCre CG Cells In Vitro

**Figure 3:**
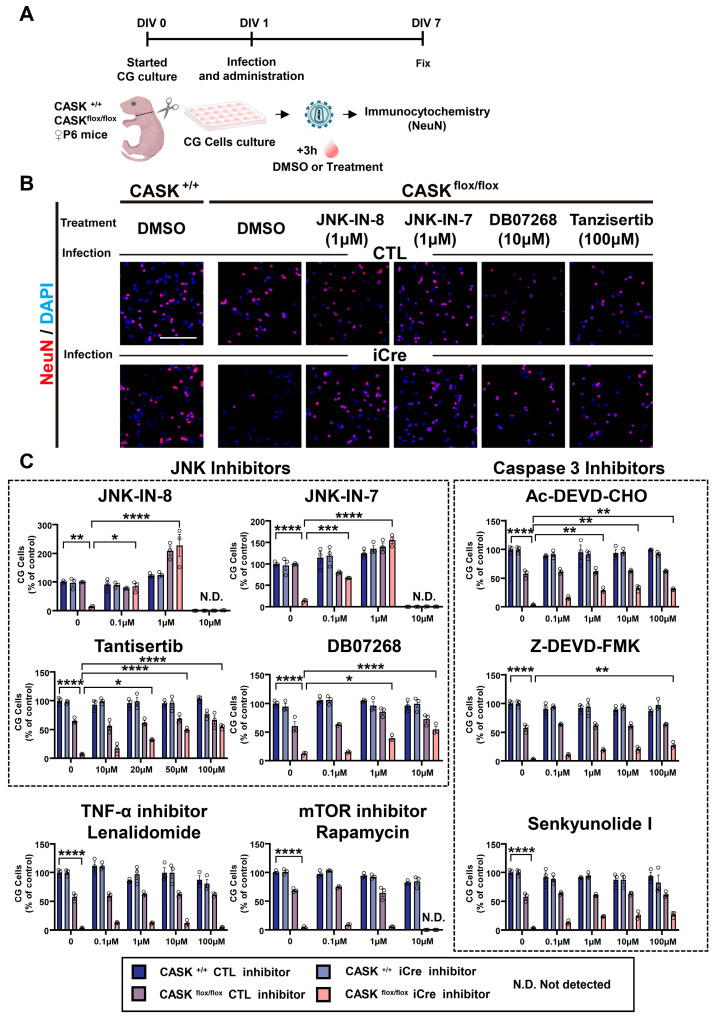
Administration of JNK inhibitors promoted the In Vitro survival rate in CG cells. (**A**). Experimental design of the intracellular signaling inhibitors administration on CG cells. CG cells were infected with lentivirus-iCre and lentivirus-CTL in DIV1, and 3 h later, the inhibitors were administered. In DIV 7, the CG cells were fixed and stained with NeuN and DAPI. (**B**). Images of CG cells stained with NeuN and DAPI, scale bar = 100 µm. (**C**). Changes in cell survival rate calculated by the NeuN and DAPI double-positive cells. The group of CASK^+/+^ CTL CG cells with DMSO was used as the control. (*n* = 3), * indicates difference between the groups. * indicates *p* < 0.05; ** indicates *p* < 0.01; *** indicates *p* < 0.001, and **** indicates *p* < 0.0001.

To investigate the effects of JNK inhibition on CG cells, we administrated JNK inhibitors on CG cells with different concentrations and stained the cells with NeuN and DAPI to evaluate the survival rate changes (Figure 3A). Without administration, the survival rate of CASK^flox/flox^ iCre CG cells was significantly lower than the CASK^+/+^ CTL CG cells (Figure 3B). We found the administration of 1 µM of JNK-IN-8 and JNK-IN-7 significantly (*p* < 0.0001, *n* = 3) increased the survival rate of CASK^flox/flox^ iCre CG cells compared to those untreated. However, administration of 10 µM of these drugs showed cytotoxicity. The administration of Tantisertib, DB07268, and SP600125, another JNK inhibitor, increased the survival rate of CASK KO CG cells as the concentration increased. (Figure 3C and Figure S3A,B). These results indicate that the inhibition of JNK signaling promotes the survival rate in CASK^flox/flox^ iCre CG Cells.

**Figure 4:**
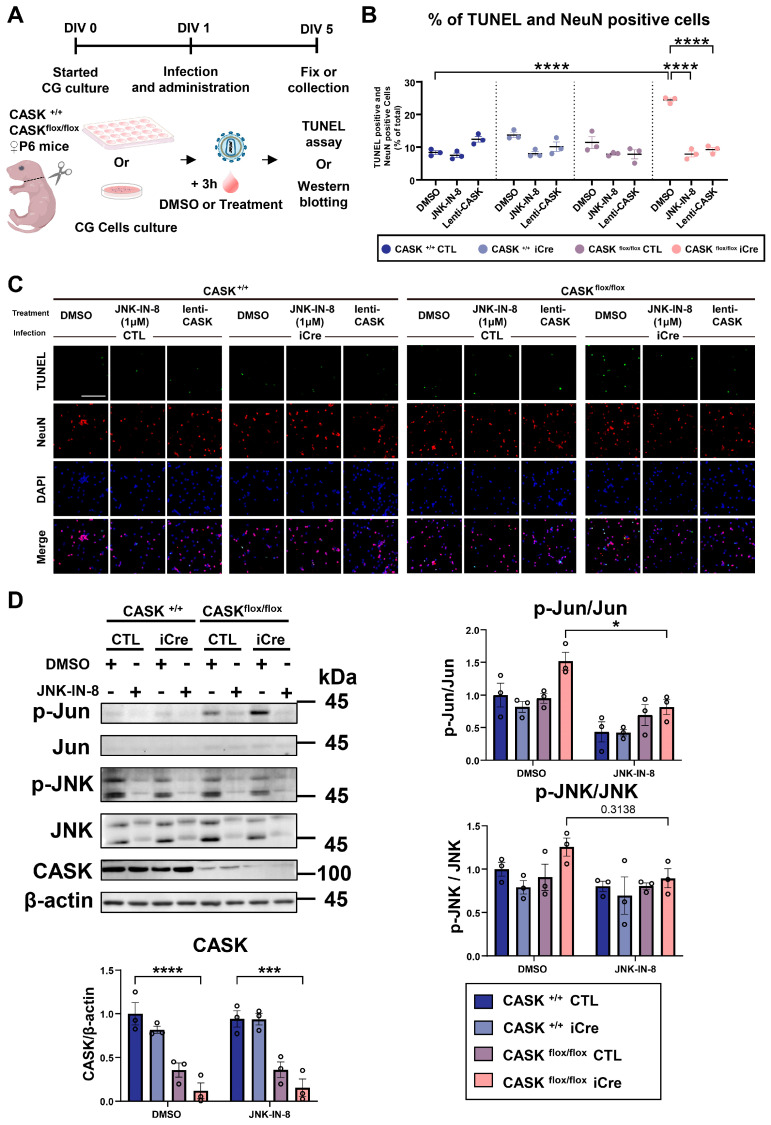
JNK inhibitor reduced apoptosis and JNK signaling in CASK^flox/flox^ iCre CG cells. (**A**). Experimental design for TUNEL assay and Western blotting. CG cells were infected with lentivirus-iCre and lentivirus-CTL in DIV1, and 3 h later, the inhibitors were administered. In DIV5, the CG cells were fixed for TUNEL assay staining or collected for Western blotting. (**B**). Percentage of TUNEL and NeuN double-positive cells. (*n* = 3). (**C**). Images of CG cells with TUNEL, NeuN, and DAPI triple staining. Green: TUNEL; Red: NeuN; Blue: DAPI, scale bar =100 µm. (**D**). Western blotting analysis on CG cells with or without JNK-IN-8 administration. (*n* = 3 in each group) * indicates difference between the groups. * indicates *p* < 0.05; *** indicates *p* < 0.001 and **** indicates *p* < 0.0001.

To verify the result, we also administered JNK signaling-related inhibitors, such as the inhibitors of Caspase 3, TNF, mTOR, and p38 signaling (Figure 3C and Figure S3A,B). Those inhibitors showed a minor effect on the survival rate of CASK-KO CG cells, but not comparable to the effect of JNK inhibitors. These indicate that the JNK inhibition has the strongest relationship with CG cell death caused by CASK deletion. JNK-IN-8 showed the strongest rescue effects on CASK-deficient CG cell survival among the JNK inhibitors we tested. To investigate the effect on apoptosis, we administered the JNK-IN-8 on CG cells and performed TUNEL staining (Figure 4A). We found that the number of TUNEL and NeuN double-positive cells was significantly (*p* < 0.0001) increased in CASK^flox/flox^ iCre CG cells compared to CASK^+/+^ CTL CG cells (Figure 4B, C). After the administration of JNK-IN-8, the number of double-positive cells was significantly (*p* < 0.0001) reduced in CASK^flox/flox^ iCre CG cells. As expected, the number of TUNEL and NeuN double-positive cells was decreased in CASK^flox/flox^ iCre CG cells after the infection with Lenti-CASK (*p* < 0.0001). The result of TUNEL indicated that the administration of JNK-IN-8 promoted the survival rate in CASK^flox/flox^ iCre CG cells, by preventing the apoptosis caused by the deletion of CASK.

We further verified the inhibition effect of JNK-IN-8 on JNK signaling in CG cells (Figure 4D). The p-c-Jun/c-Jun (*p* < 0.05, *n* = 3) and p-JNK/JNK (*p* = 0.3138, *n* = 3) were suppressed by the JNK-IN-8 administration. We also tested the effect of Lenti-CASK infection on CASK^+/+^ CG cells. Although Lenti-CASK infection reduced p-JNK/JNK compared to the uninfected control, the effect was smaller than that of the JNK-IN-8 administration (Figure S3C).

### 3.4. Gene Expression Analyses Indicate That JNK Inhibition Specifically Reduced Apoptosis in CASK^flox/flox^ iCre CG Cells

**Figure 5:**
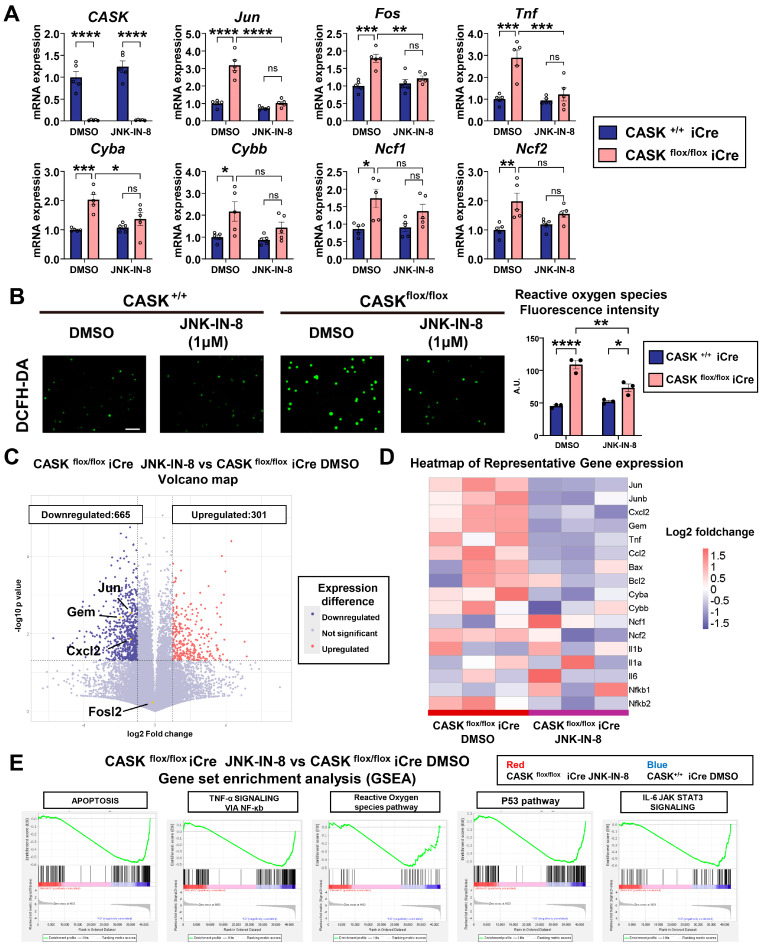
JNK inhibitor reduced apoptosis and the reactive oxygen species pathway. (**A**). Realtime-qPCR analysis on JNK-IN-8 or DMSO-treated CG cells. (*n* = 5 in each group). (**B**). Reactive oxygen species visualization and quantification of fluorescence intensity, scale bar =100 µm. (*n* = 3). (**C**). Volcano map of gene expression difference between JNK-IN-8 treated CASK^flox/flox^ iCre CG cells and DMSO-treated CASK^flox/flox^ iCre CG cells. Red indicated genes upregulated in JNK-IN-8 treated CG cells, blue indicated genes downregulated in JNK-IN-8 treated CG cells. Detected by RNA-seq. (Threshold: *p* < 0.05, Fold change over 2). (**D**). Heatmap of the representative gene expression detected by RNA-seq. (**E**). Representative Gene Set Enrichment Analysis (GESA) results of apoptosis-related pathways.* indicates difference between the groups. * indicates *p* < 0.05; ** indicates *p* < 0.01; *** indicates *p* < 0.001; and **** indicates *p* < 0.0001. ns indicates no significant difference.

To further confirm the JNK inhibitor’s effect on the alteration of the gene expression in CG cells, we examined the expression levels of those genes in JNK-IN-8- or DMSO-treated CG cells by realtime-qPCR (Figure 5A and Figure S4). We found that the expression levels of c-Jun (*p* < 0.0001, *n* = 5), c-Fos (*p* < 0.01, *n* = 5), and Tnf (*p* < 0.001, *n* = 5) were significantly reduced after the administration of JNK-IN-8 in CASK^flox/flox^ iCre CG cells. In addition, the levels of ROS (*p* < 0.01, *n* = 3) and ROS-related genes were also reduced by the administration of JNK-IN-8 in CASK^flox/flox^ iCre CG cells (Figure 5A,B). By RNA-seq analysis, we investigated the transcriptomic differences between JNK-IN-8–treated and DMSO-treated CASK^flox/flox^ iCre CG cells and identified 301 significantly upregulated and 665 significantly downregulated genes in the JNK-IN-8–treated group (Figure 5C). We also found that apoptosis genes such as Gem and Cxcl2 were significantly reduced in JNK-IN-8 treated CASK^flox/flox^ iCre CG cells compared to those in DMSO-treated CASK^flox/flox^ iCre CG cells (Figure 5C,D). From GESA (Figure 5E), cell death-related signaling, such as apoptosis and ROS pathway, was suppressed by the administration of JNK-IN-8. In addition, inflammation-related pathways—including TNF-α signaling, p53 signaling, and IL-6–JAK–STAT3 signaling—were also significantly suppressed by JNK-IN-8 treatment (Figure 5E). However, the other apoptosis signaling-related genes, such as Bax and Bcl2, were not significantly affected by the administration of JNK-IN-8 (Figure 5D), which was also verified by realtime-qPCR (Figure S4), indicating that JNK signaling plays a specific role in suppressing apoptosis and ROS caused by CASK deletion. By inhibiting JNK signaling, CG cell survival was improved in CASK-deficient CG cells by suppressing the expression of apoptotic genes In Vitro. This indicates application of JNK inhibitors may be the method for preventing cerebellar hypoplasia in MICPCH syndrome.

### 3.5. The Administration of the JNK Inhibitor In Vivo Shows Preventive Effect Against Neurodegeneration

**Figure 6:**
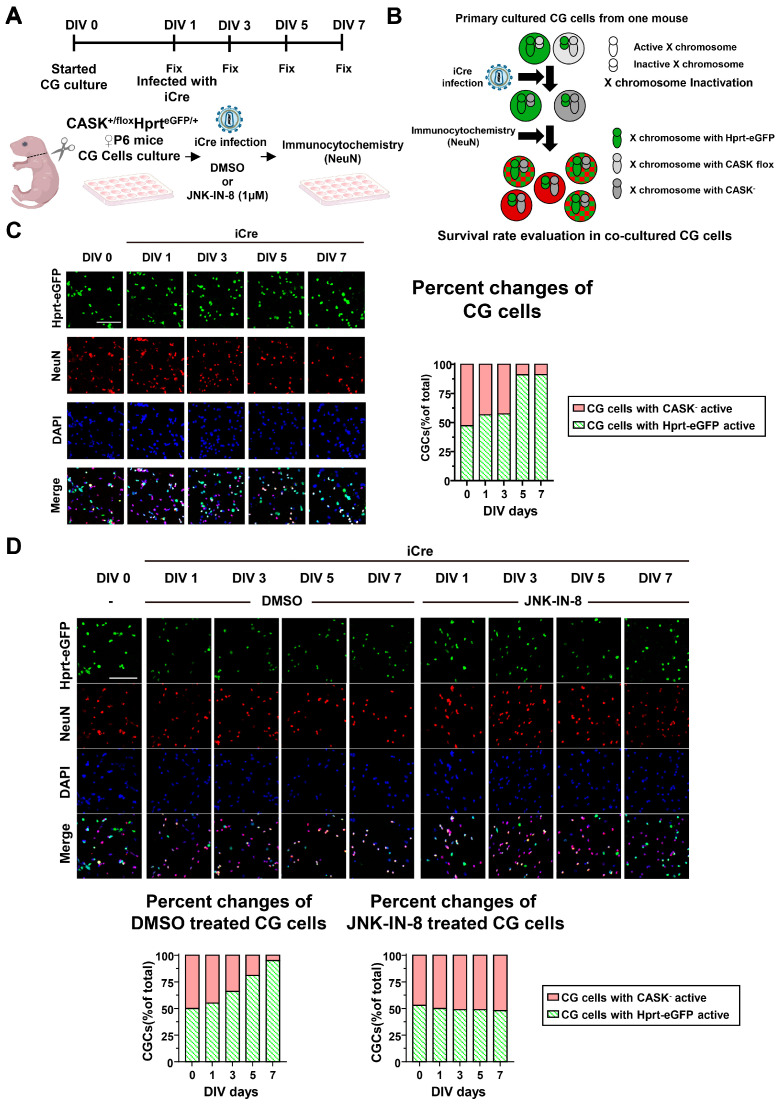
Survival rate evaluation on heterozygous CASK knockout CG cells. (**A**). Experimental design on CASK^+/flox^Hprt^eGFP/+^ CG cells. (**B**). Schematic diagram showing how survival rate evaluation was conducted on different X chromosome-activated CG cells in co-cultivation. And the CG cells with double positive of eGFP and NeuN were viewed as the CG cells with Hprt-eGFP X chromosome active. The NeuN-positive CG cells without eGFP fluorescent light were viewed as the CG cells with CASK knockout (CASK^-^), X chromosome active. (**C**). Immunocytochemistry-stained Images of eGFP, NeuN, and DAPI in CG cells. The percent changes in CG cells were evaluated by those images. Green: Hprt-eGFP; Red: NeuN; Blue: DAPI. scale bar = 100 µm. (*n* = 3). (**D**). Immunocytochemistry stained Images of eGFP, NeuN, and DAPI in JNK-IN-8 or DMSO administrated CG cells. The percent changes in CG cells were evaluated. Green: Hprt-eGFP; Red: NeuN; Blue: DAPI. scale bar = 100 µm. (*n* = 3).

Due to the perinatal lethality of CASK knockout, we evaluated the effect of JNK inhibition on the cerebellar hypoplasia In Vivo in female CASK heterozygote knockout mice, whose genetic background is representative of the majority of MICPCH syndrome. Our previous study demonstrated that X-chromosome inactivation produced a mosaic distribution of the CASK-expressing and CASK-non-expressing neurons in the brain of female CASK heterozygote knockout mice [8]. To investigate whether the CG cell death in female CASK heterozygote knockout mice is specific to CASK knockout cells or not, we utilized Hprt-eGFP mice to visualize the pattern of X-chromosome inactivation. In Hprt-eGFP mice, the eGFP expression cassette under the control of the CAG promoter was knocked into the hprt locus on the X-chromosome [21]. We generated CASK flox and Hprt-eGFP trans-heterozygote mice (CASK^flox^; Hprt^eGFP^) by crossing CASK flox and Hprt-eGFP mice. In these mice, the cells expressing eGFP indicate that the X-chromosome inherited from Hprt-eGFP mice is intact and expresses wild-type CASK. In contrast, the cells that are not expressing eGFP indicate which X-chromosome inherited from Hprt-eGFP is inactivated and has an active X-chromosome with the CASK flox allele. We prepared CG cell culture from CASK^flox^; Hprt^eGFP^ mice and infected them with lentivirus expressing iCre to create CASK KO and CASK intact culture (Figure S5). We examined the survival rate by NeuN and DAPI staining (Figure 6A,B). By analyzing immunocytochemistry-stained images, we found that the percentage of the CG cells without eGFP expression is gradually reduced in these cultures, indicating that CASK knockout cells die selectively without affecting CASK-intact cells (Figure 6C). We administered JNK-IN-8 or DMSO in the co-cultured CG cells 3 h after the iCre infection. We found the DMSO administration did not increase the percentage of CASK knockout CG cells (Figure 6D). In contrast, JNK-IN-8 administration rescued the reduction in CASK knockout cells, and the ratio between eGFP-positive and negative cells was almost 1:1 (Figure 6D). These results suggest that apoptosis occurs selectively in CASK knockout CG cells and can be prevented by the administration of JNK-IN-8.

**Figure 7:**
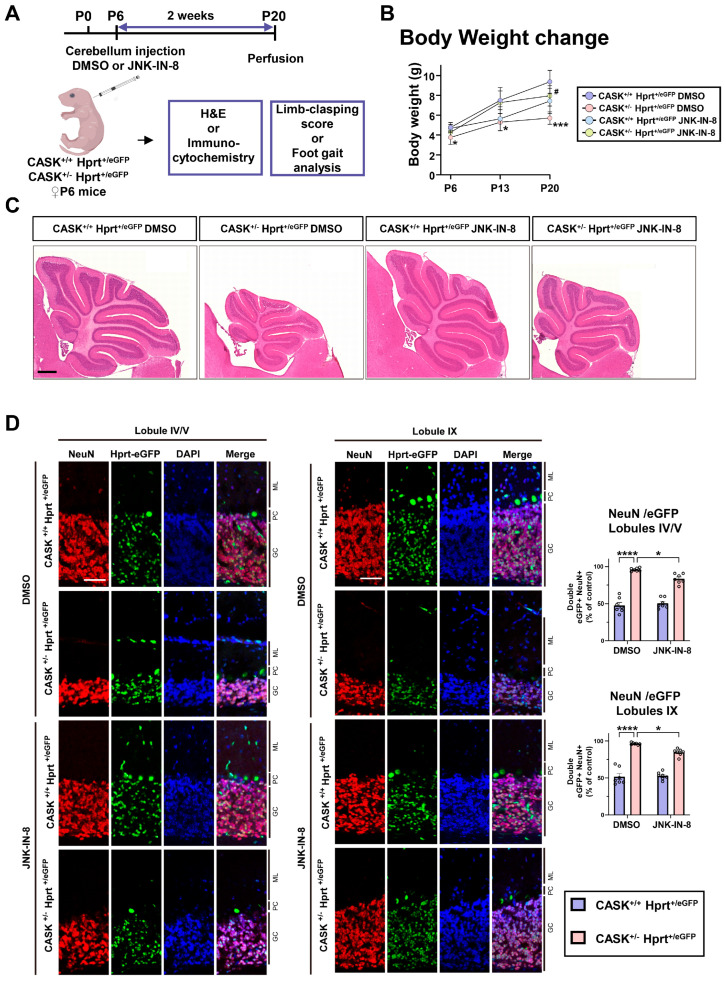
JNK-IN-8 administration In Vivo cerebellum development of CASK^+/-^ HprteGFP^/+^ mice. (**A**). Experimental design on CASK^+/-^ Hprt^eGFP/+^ and CASK^+/-^ Hprt^eGFP/+^ mice. (**B**). Body Weight changes in CASK^+/-^ Hprt^eGFP/+^ and CASK^+/-^ Hprt^eGFP/+^ mice. (*n* = 5)(* indicates difference between CASK^+/-^ Hprt^+/eGFP^ DMSO and CASK^+/+^ Hprt^+/eGFP^ DMSO, # indicates difference between CASK^+/-^ Hprt^+/eGFP^ JNK-IN-8 and CASK^+/-^ Hprt^+/eGFP^ DMSO). (**C**). Photography of H&E stained cerebellum slices, scale bar = 500 µm. (**D**). Immunohistochemistry stained Lobules IV/V slices and Lobules IX. ML, molecular layer; PC, Purkinje cell layer; GC, granular cell layer. The ratio of double-positive cells of NeuN and eGFP was calculated. (*n* = 7) * indicates difference between the groups. *, ^#^ indicate *p* < 0.05; *** indicates *p* < 0.001; and **** indicates *p* < 0.0001.

Next, to evaluate the effect of JNK-IN-8 on heterozygous CASK knockout mice In Vivo, we administered JNK-IN-8 in the cerebellum of P6 CASK^+/-^ Hprt^eGFP/+^ and CASK^+/+^ Hprt^eGFP/+^ mice and analyzed 2 weeks after the injection (Figure 7A). Before injection, the body weight of CASK^+/-^ Hprt^eGFP/+^ mice was significantly (P6: *p* < 0.05; P13: *p* < 0.05; P20: *p* < 0.001, *n* = 5 each genotype) lower than CASK^+/+^ Hprt^eGFP/+^ mice (Figure 7B and Figure S6A). The JNK-IN-8 administration significantly (*p* < 0.05) increased the body weight of CASK^+/-^ Hprt^eGFP/+^ mice, 2 weeks after the injection. In H&E-stained cerebellum slices, we found that the size of the cerebellum in CASK^+/-^ Hprt^eGFP/+^ mice was smaller than that of CASK^+/+^ Hprt^eGFP/+^ mice at P20. The administration of JNK-IN-8 increased the size in CASK^+/-^ Hprt^eGFP/+^ mice (Figure 7C). Moreover, by immunohistochemistry staining and H&E staining on slices from Lobules IX and Lobules IV/V, we found the granular cell (GC) layer of the CASK^+/-^ Hprt^eGFP/+^ was thinner and the cell density was lower than that of CASK^+/+^ Hprt^eGFP/+^ (Figure 7D and Figure S6B). In the GC layer, nearly 100% of the cells were double-positive for NeuN and eGFP in DMSO-treated CASK^+/-^ HprteGFP^/+^ mice, in contrast to approximately 50% in CASK^+/+^ Hprt^eGFP/+^ mice in both lobules IV/V and IX (Figure 7D). This finding indicates that CASK-deficient CG cells in CASK^+/-^ Hprt^eGFP/+^ mice underwent cell death, and only CASK-intact, eGFP-expressing cells survived. Following JNK-IN-8 administration, the proportion of NeuN and eGFP double-positive cells was significantly reduced (*p* < 0.05, *n* = 7) in both lobules IV/V and IX, suggesting that some CASK-deficient CG cells were rescued in these regions. A similar trend was observed in the Purkinje cell (PC) layer, where most cells in CASK^+/-^ Hprt^eGFP/+^ mice were double-positive for NeuN and eGFP. After JNK-IN-8 administration, the proportion of double-positive PCs decreased (*p* = 0.1273 in lobules IV/V; *p* = 0.1657 in lobules IX; *n* = 7 each) (Figure S7). These morphological results indicated that JNK-IN-8 administration In Vivo prevents cerebellar cell loss in CASK^+/-^ Hprt^eGFP/+^ mice.

**Figure 8:**
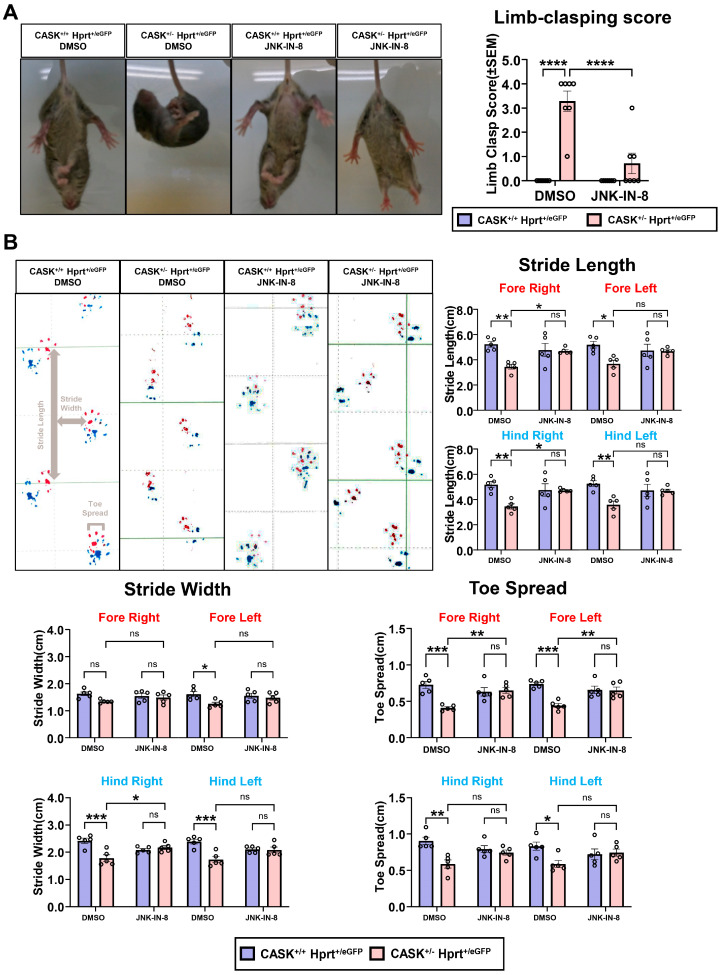
Behavioral analyses indicated that JNK-IN-8’s administration improved cerebellum function. (**A**). Photography on P20 CASK^+/+^ Hprt^eGFP/+^ and CASK^+/-^ Hprt^eGFP/+^ mice, treated with or without JNK-IN-8. Limb-clasping scores were calculated by their behavioral performance. (*n* = 7). (**B**). Gait analysis record and the stride length, stride width, and toe spread were measured separately from each limb. Red ink: Forelimbs; Blue ink: Hindlimbs. (*n* = 5) * indicates difference between the groups. * indicates *p* < 0.05; ** indicates *p* < 0.01; *** indicates *p* < 0.001; and **** indicates *p* < 0.0001. ns indicates no significant difference.

Lastly, we examined the effect of the administration of the JNK inhibitor on the behavior of CASK^+/-^ Hprt^eGFP/+^ mice. We performed a limb clasping test on these mice that monitor cerebellar ataxia. CASK^+/-^ Hprt^eGFP/+^ mice exhibited clasping and crossing of their limbs and were immobilized indicating they have cerebellar ataxia (Figure 8A). However, after the JNK-IN-8 injection into cerebellum at P6, the mobility was improved, and the limb-clasping score was significantly decreased (*p* < 0.0001, *n* = 7 each) compared to untreated mice. We also performed gait analysis on these mice. We found the stride length (Fore Right, Hind Right, and Hind Left: *p* < 0.01; Fore Left: *p* < 0.05, *n* = 5 each) and toe spread (Fore Right and Fore Left: *p* < 0.001; Hind Right: *p* < 0.01; Hind Left: *p* < 0.05, *n* = 5 each) of CASK^+/-^ Hprt^eGFP/+^ mice were significantly shorter than CASK^+/+^ Hprt^eGFP/+^ (Figure 8B). These behavioral analyses indicated that the cerebellar function of CASK^+/-^ Hprt^eGFP/+^ mice was improved by the administration of JNK-IN-8.

## 4. Discussion

Although apoptosis caused by CASK deletion has been well documented in previous neurological studies [2,22,23], the specific molecular pathways mediating CG cell death have remained largely unexplored. Our study is the first to perform bulk RNA-seq and pathway-level analysis on cerebellar granule (CG) cells from CASK^flox/flox^ iCre mice, revealing that CASK deficiency induces significant activation of the JNK signaling and reactive oxygen species (ROS) pathways. This result was further confirmed by qPCR, Western blotting, and TUNEL assays, supporting the hypothesis that CASK plays a crucial role in maintaining cellular homeostasis in the cerebellum.

Our findings are consistent with previous reports linking JNK activation and ROS accumulation to neurodegenerative processes [24,25]. The upregulation of apoptosis-related genes such as c-Jun, Fosl2, Gem, and Cxcl2, alongside the increased phosphorylation of JNK and c-Jun, strongly suggests that the JNK pathway is a key effector of CG cell death in the absence of CASK. Importantly, JNK-IN-8 treatment effectively suppressed both the JNK phosphorylation and the transcriptional activation of ROS-related genes, thereby rescuing cell survival. Among the JNK inhibitors tested, JNK-IN-8 showed the most robust and selective effect, highlighting its potential as a therapeutic compound for CASK-related neurodegeneration.

Recent studies have continued to elucidate the multifaceted roles of CASK in neural development and disease. For instance, Patel et al. (2022) reported that complete loss of CASK leads to profound cerebellar degeneration in both humans and mice, yet the specific mechanisms driving cell loss remained unclear [26]. Moreover, Tibbe et al. (2021) characterized alternative CASK transcripts in the human brain, suggesting isoform-specific functional diversity that may contribute to regional vulnerability [27]. While the developmental consequences of CASK mutations are well-documented, therapeutic strategies targeting downstream effectors of CASK loss are still largely unexplored.

Our study uniquely identifies the JNK-ROS signaling axis as a critical mediator of CG cell apoptosis in CASK-deficient mice, advancing the field by providing the first In Vivo evidence that targeted inhibition of JNK signaling can alleviate both structural and functional cerebellar deficits in a genetically relevant model of MICPCH syndrome. Previous work has implicated JNK activation in various neurodegenerative contexts [25,26,27,28,29,30,31], but its involvement in CASK-related pathology has not been directly examined until now. By leveraging both transcriptomic and functional assays, our results bridge a critical gap between CASK deficiency and its downstream pathological cascades, positioning JNK signaling as a viable therapeutic target. This mechanistic insight offers a conceptual advance beyond earlier studies that primarily focused on synaptic dysregulation or broad neurodegeneration without molecular resolution [7,8,9].

Moreover, our In Vivo experiments in heterozygous CASK knockout mice (CASK^+/-^ Hprte^GFP/+^) provided compelling evidence that JNK inhibition alleviates cerebellar hypoplasia and motor deficits. Notably, eGFP-negative cells—representing the CASK-deficient population due to X-inactivation—were selectively lost in untreated mice, but their survival was preserved following JNK-IN-8 administration. Behavioral improvements in limb-clasping and gait analysis further confirmed the functional recovery of cerebellar circuits, particularly in the hemisphere receiving JNK-IN-8 injections.

Nevertheless, our study has several limitations. First, while CG cells were the primary focus, the potential involvement of other cerebellar cell types, such as Purkinje cells and interneurons, was not fully explored. Second, our omics analysis focused primarily on apoptosis and oxidative stress pathways; other altered processes, including metabolism-related changes [32,33], may also play a role and warrant future investigation. In addition, while bulk RNA-seq provided valuable global insight, single-cell resolution would help distinguish cell-type–specific responses and mosaic expression patterns due to X-inactivation. Further studies utilizing single-cell RNA-seq or spatial transcriptomics would be particularly informative.

Lastly, while our results strongly support the use of JNK inhibitors to rescue CASK-dependent phenotypes, the long-term safety and efficacy of such compounds In Vivo remain to be established. The possibility of off-target effects or compensation by parallel pathways cannot be excluded. Nonetheless, our work provides a molecular framework that bridges CASK deficiency and cell death signaling in the cerebellum and opens the door for targeted therapeutic strategies in MICPCH syndrome.

## 5. Conclusions

Our study demonstrates that CASK deletion in cerebellar granule cells leads to apoptosis and neurodegeneration through activation of the JNK signaling and ROS pathways. Using RNA-seq, molecular assays, and behavioral analysis In Vitro and In Vivo, we showed that pharmacological inhibition of JNK signaling—particularly via JNK-IN-8—effectively suppresses CG cell death and alleviates cerebellar ataxia in a mouse model of MICPCH syndrome. These findings identify the JNK pathway as a critical mediator of CASK-related cerebellar pathology and support the potential utility of JNK inhibitors as therapeutic agents for neurodevelopmental disorders involving CASK dysfunction.

## Data Availability

The RNA-seq data and other raw datasets are available from Q.G. upon request (gq_2222@126.com). Materials used in the study are available from K.T. upon request (ktabuchi@shinshu-u.ac.jp).

## Supplementary Materials

The following supporting information can be downloaded at: https://www.mdpi.com/article/10.3390/cells14100750/s1, Figure S1: Male homozygous CASK KO (CASK^-/Y^) mice showed developmental delay cerebellum morphological abnormality; Figure S2: Additional results of transcription difference between CASK^flox/flox^ iCre and CASK^+/+^ iCre cerebellum granule (CG) cells; Figure S3: JNK, p38 inhibitor may improve CASK^flox/flox^ iCre CG cells survival rates; Figure S4: Additional results of realtime-qPCR analysis; Figure S5: Breeding information of heterozygous CASK KO with Hprt-eGFP (CASK^+/-^ Hprt^eGFP/+^) mice; Figure S6: Heterozygous CASK KO with Hprt-eGFP (CASK^+/-^ Hprt^eGFP/+^) mice showed cerebellar developmental disorders; Figure S7: JNK-IN-8 reduced the ratio of Calbindin /eGFP double positive cells in Purkinje cell layer minorly; Figure S8: Summary of the study; Table S1: oligo primers for q-PCR; Table S2: information on antibodies used in this study; Table S3: TPM expression matrix; Supplementary Methods.
