# Supplementary material for: Jun N-Terminal Kinase Inhibitor Suppresses CASK Deficiency-Induced Cerebellar Granular Cell Death in MICPCH Syndrome Model Mice
Source: Cells. 2025 May 20;14(10):750. doi: 10.3390/cells14100750 (PMC12109623; doi:10.3390/cells14100750)

CASK$^{+/+}$ and CASK $^{flox/flox}$ Repeat group 1

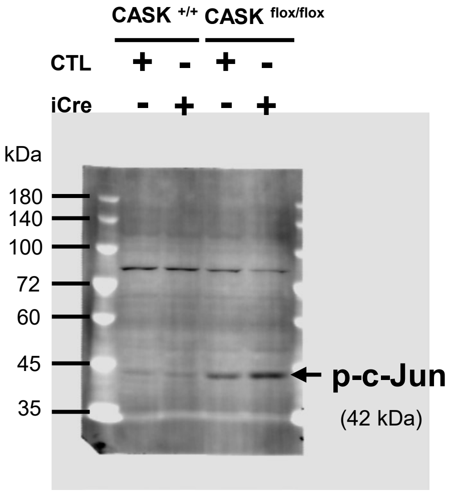

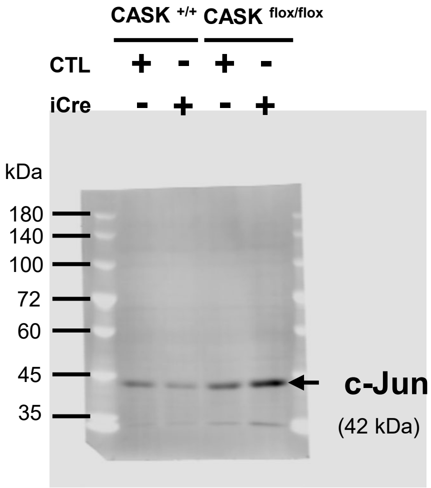

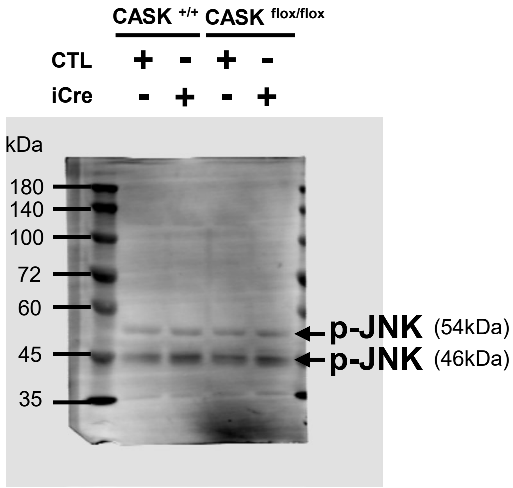

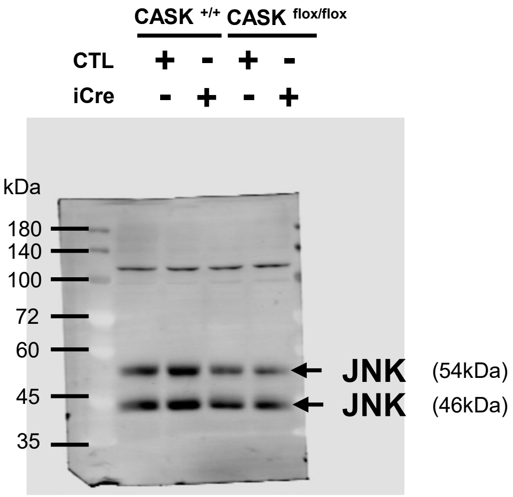

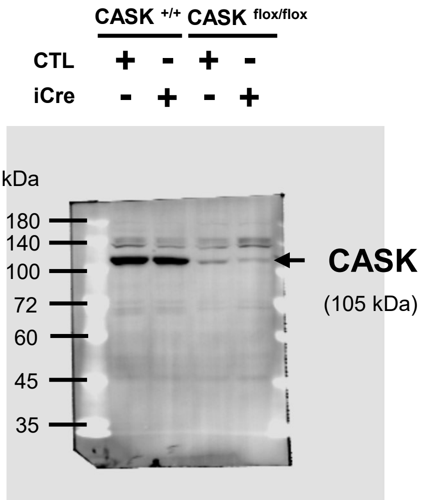

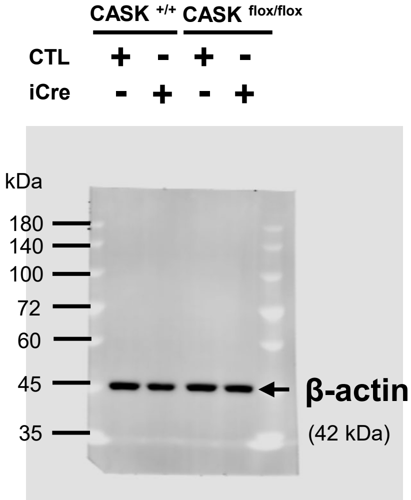

CASK$^{+/+}$ and CASK $^{flox/flox}$ Repeat group 2

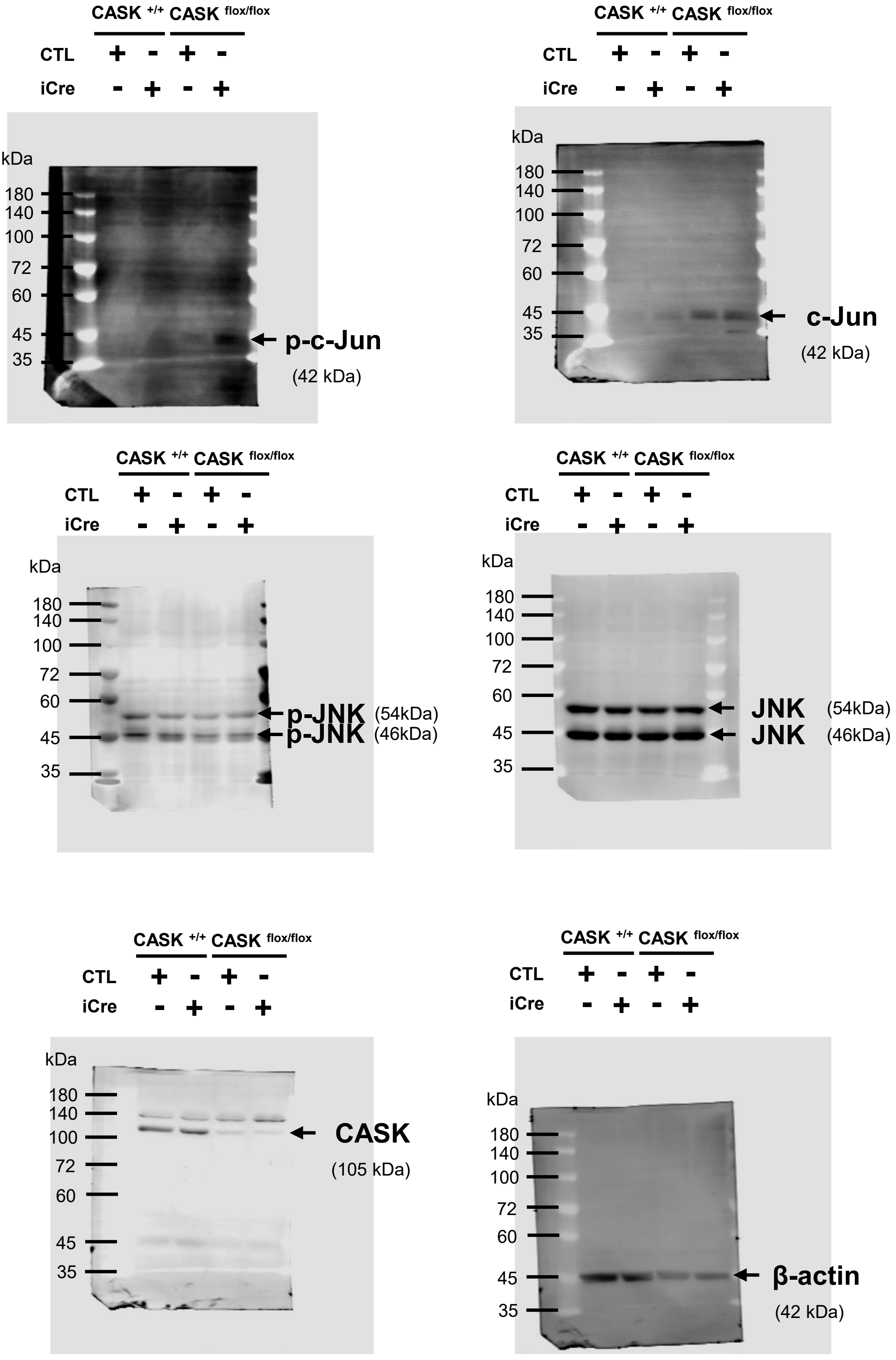

CASK$^{+/+}$ and CASK $^{flox/flox}$ Repeat group 3

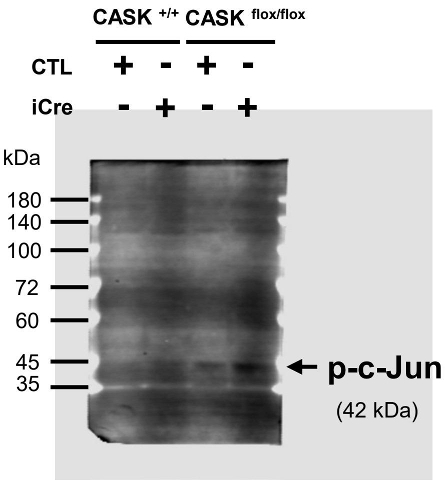

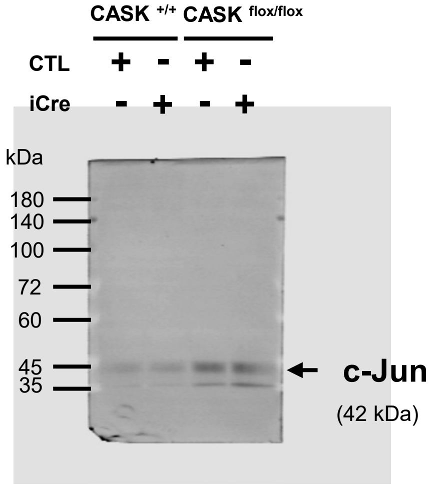

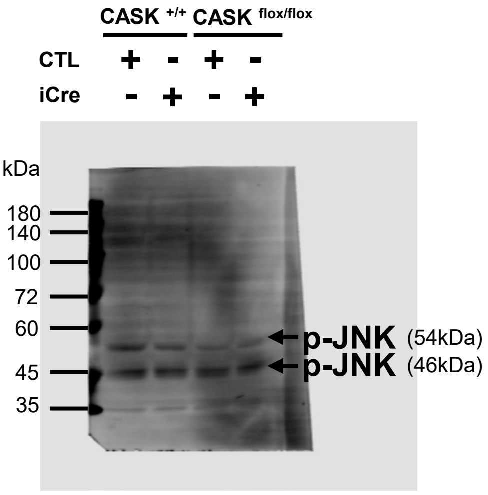

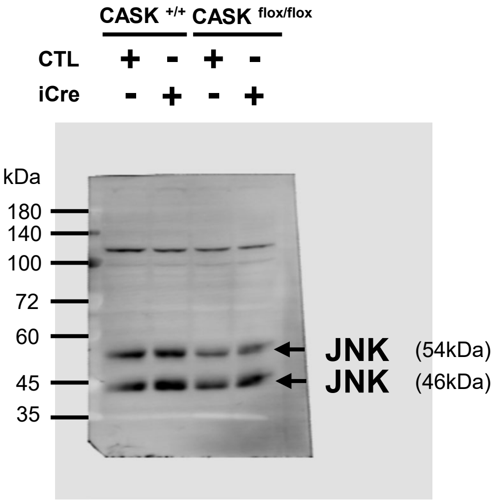

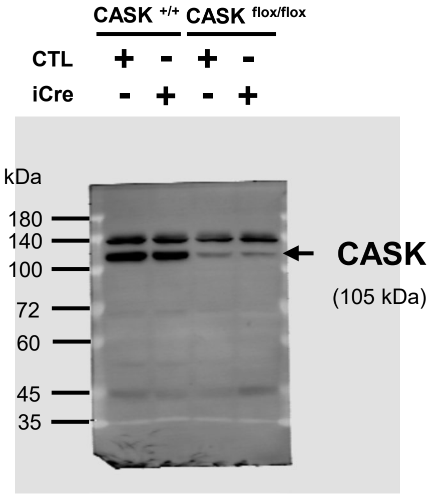

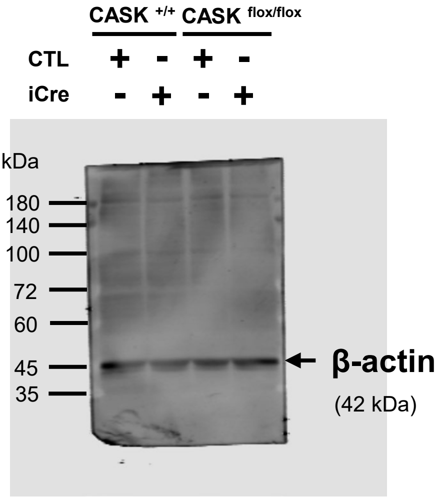

CASK$^{+/+}$ and CASK $^{flox/flox}$ Repeat group 1
Treatment DMSO or JNK-IN-8

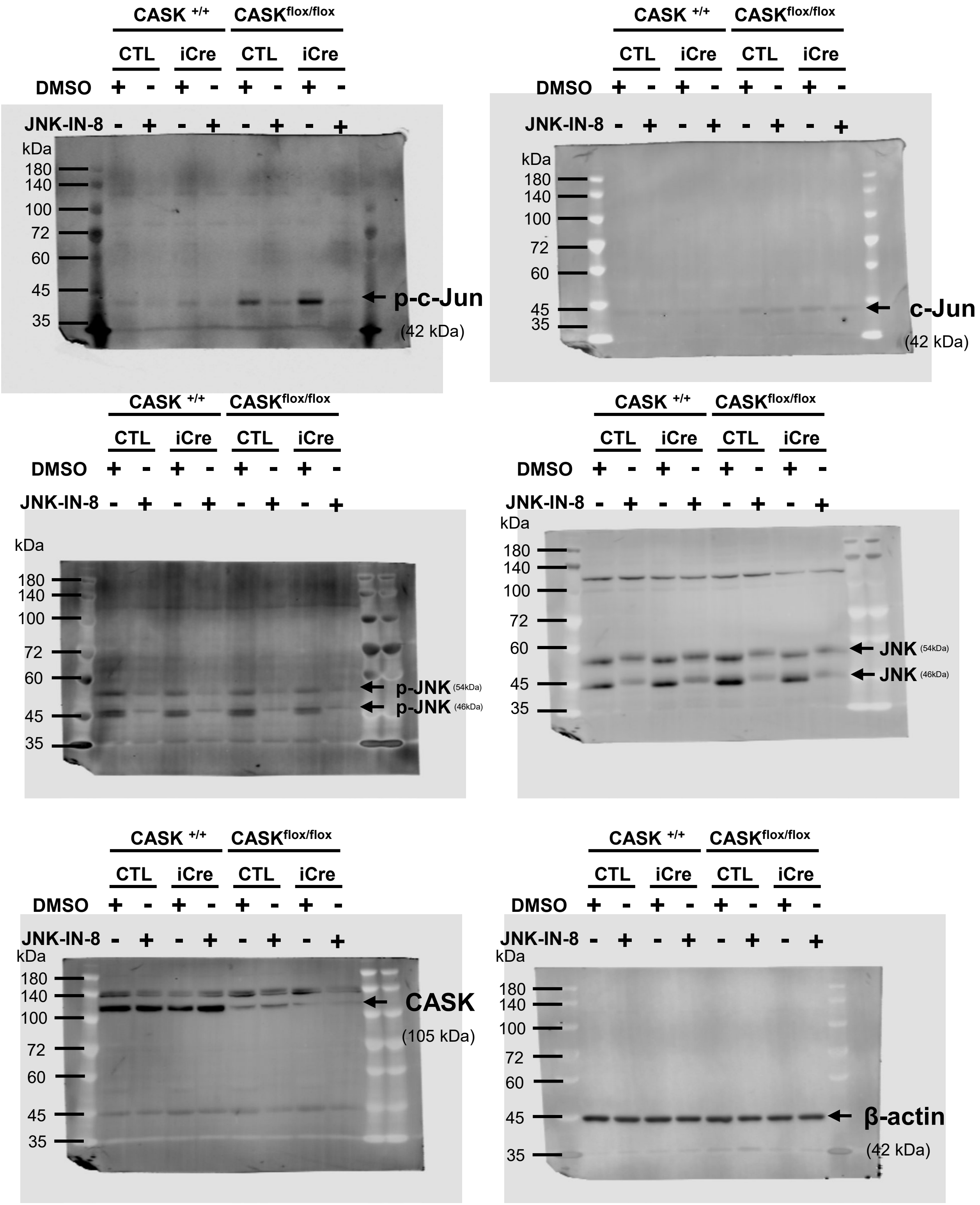

CASK+/+ and CASK flox/flox Repeat group 2
Treatment DMSO or JNK-IN-8

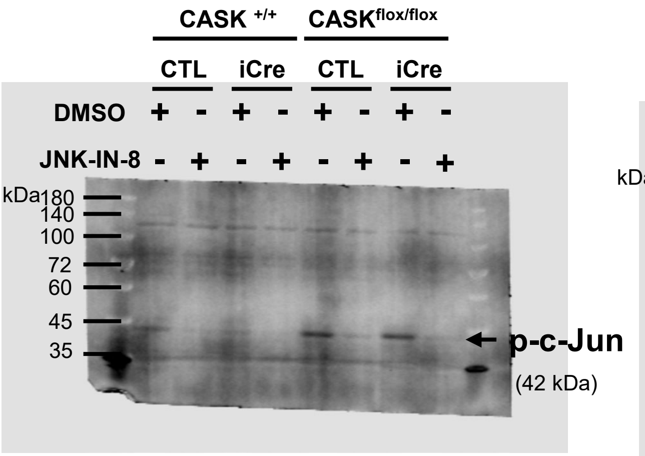

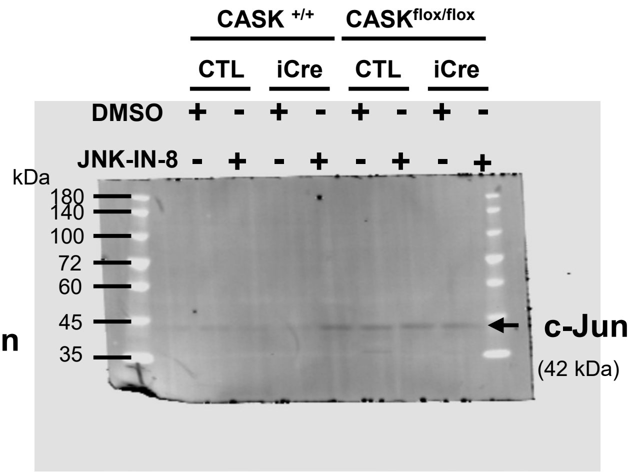

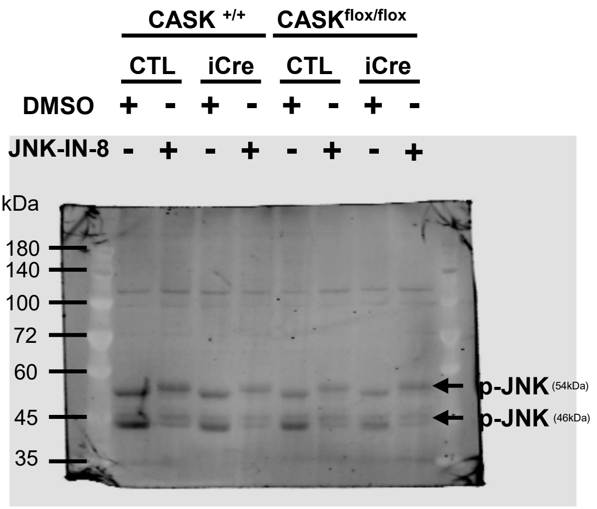

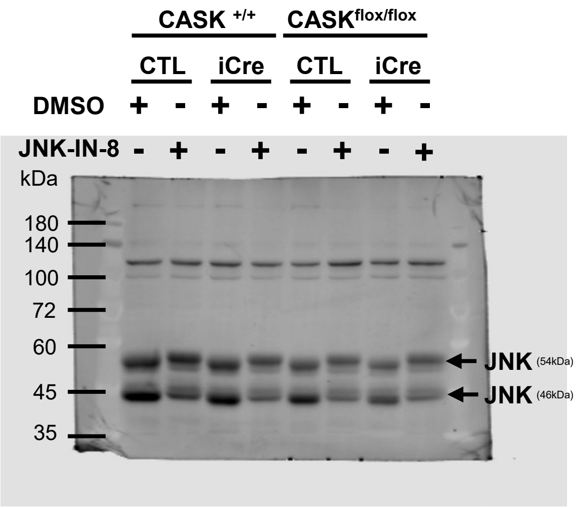

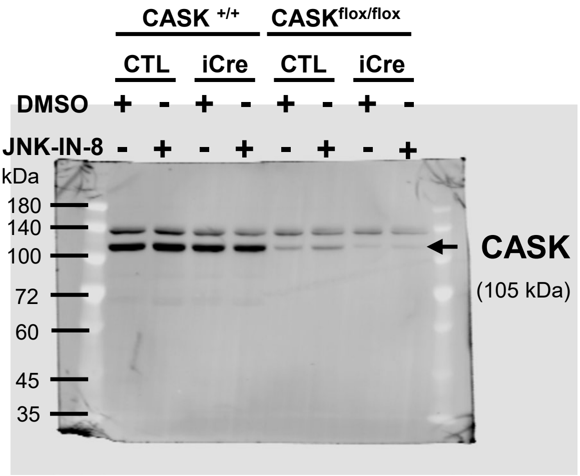

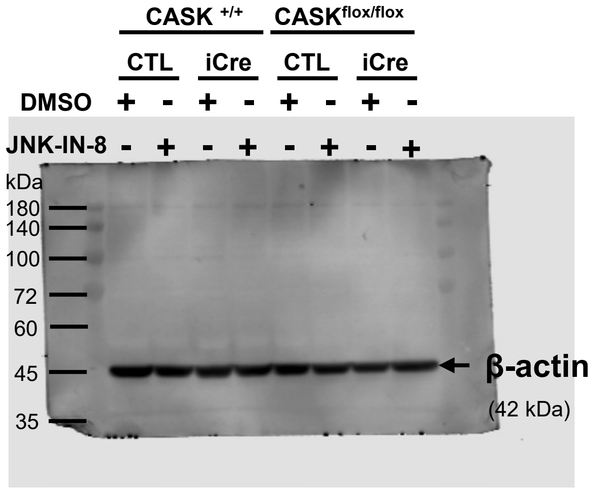

CASK$^{+/+}$ and CASK $^{flox/flox}$ Repeat group 3 Treatment DMSO or JNK-IN-8

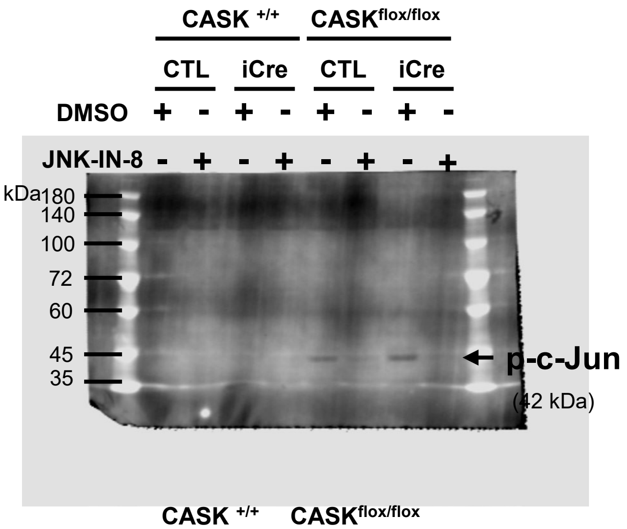

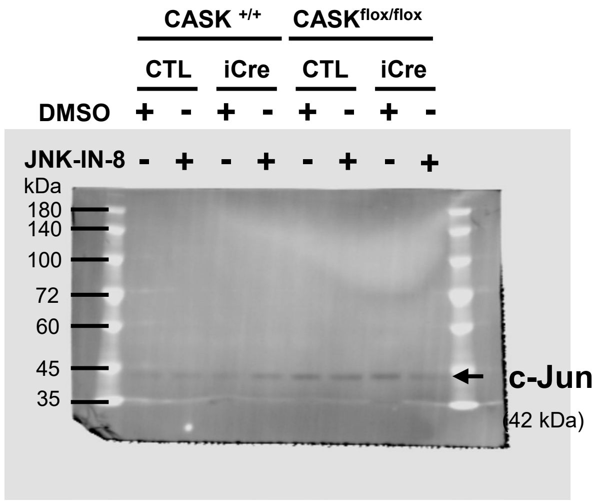

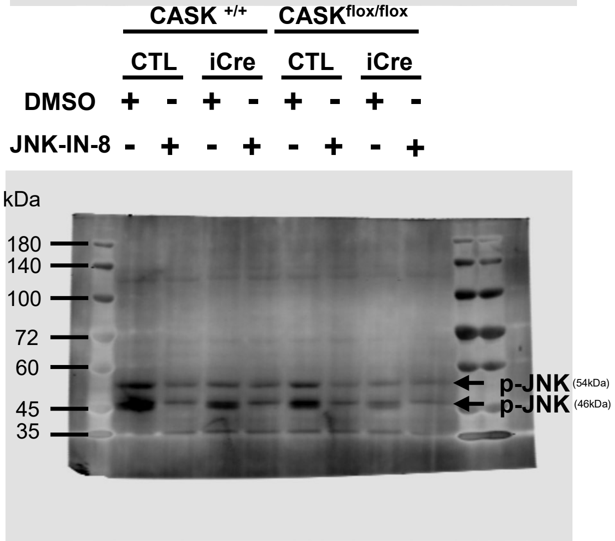

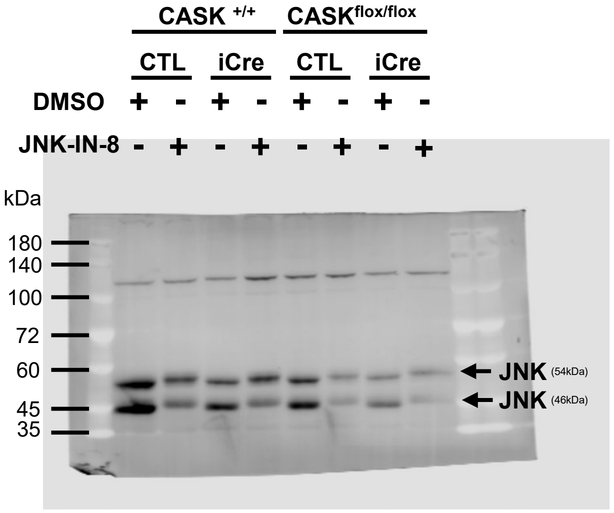

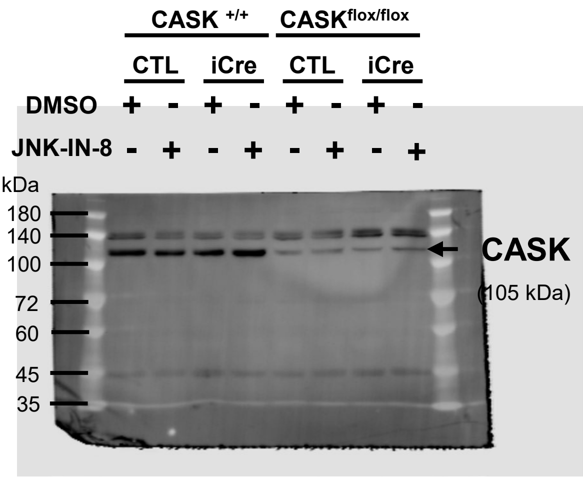

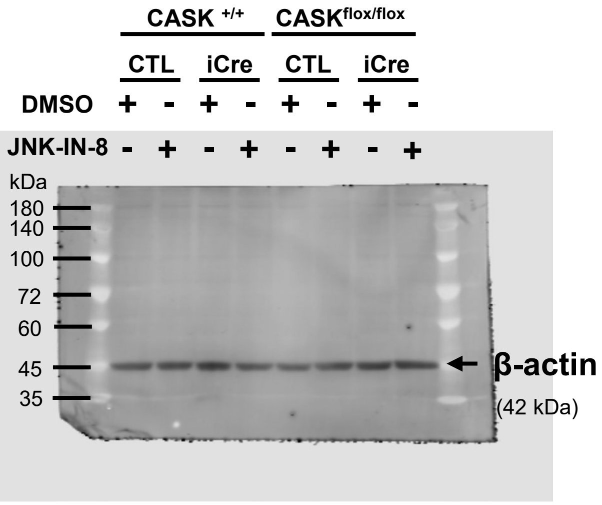

WT overexpression Repeat group 1-3

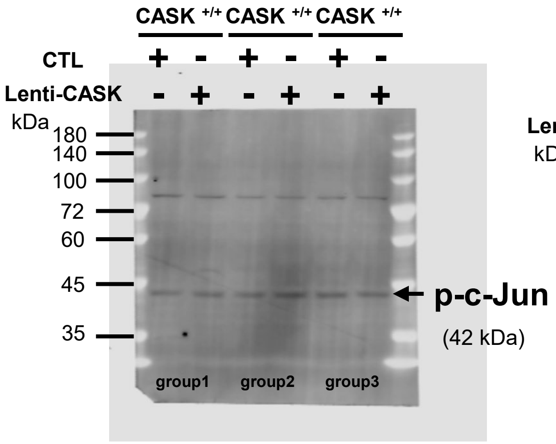

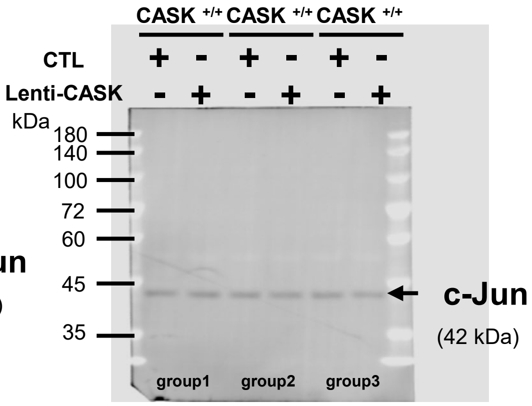

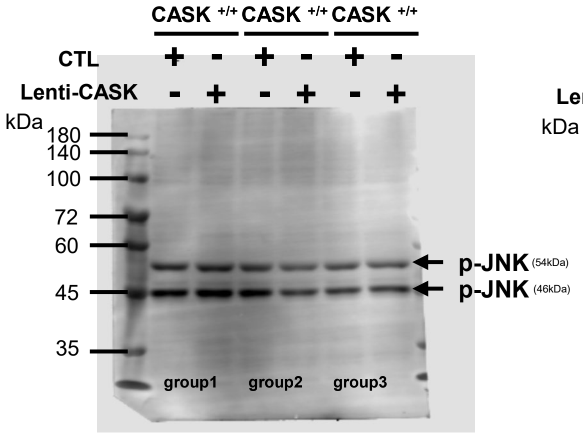

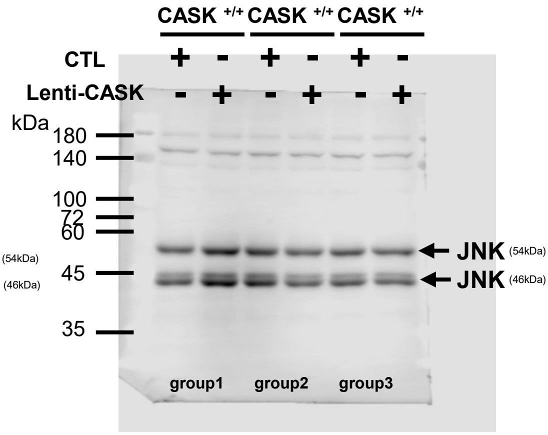

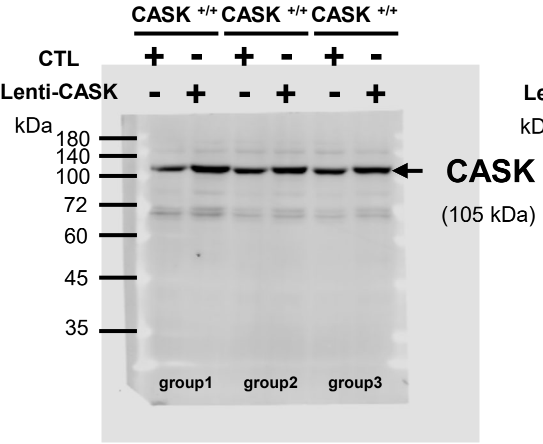

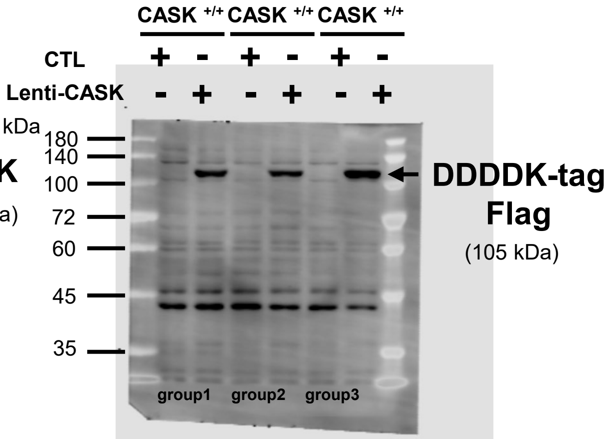

WT overexpression Repeat group 1-3

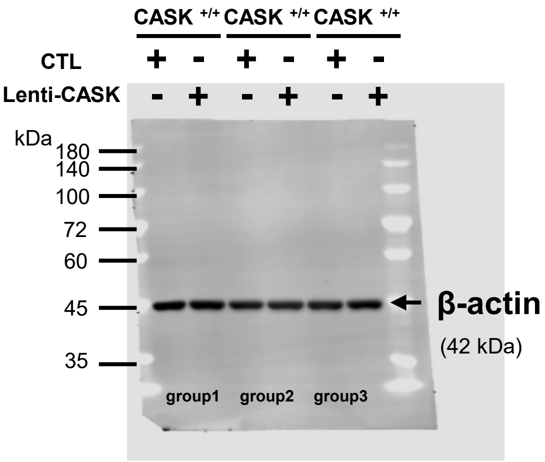

Supplement: Supplementary file 1 [file cells-14-00750-s001.zip › Original Data1.pdf]
