# Supplementary material for: Jun N-Terminal Kinase Inhibitor Suppresses CASK Deficiency-Induced Cerebellar Granular Cell Death in MICPCH Syndrome Model Mice
Source: Cells. 2025 May 20;14(10):750. doi: 10.3390/cells14100750 (PMC12109623; doi:10.3390/cells14100750)
Supplement: Supplementary file 1 [file cells-14-00750-s001.zip › Supplement figures 20250514.pdf]

Figure S1

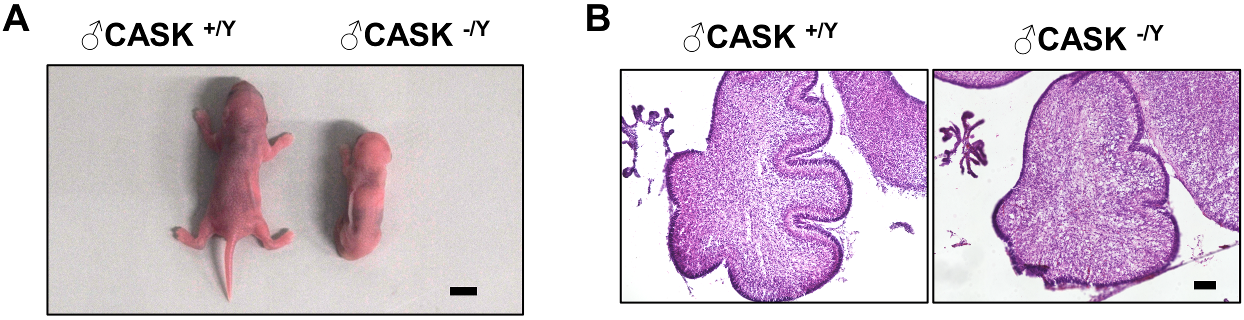

Supplementary Figure S1: Male homozygous CASK KO ($CASK^{-/Y}$) mice showed developmental delay cerebellum morphological abnormality

A. Photos of P0 male CASK $^{+/Y}$ and male CASK $^{-/Y}$ mice.

B. H&E stained cerebellum slice from male CASK $^{+/Y}$ and male CASK $^{-/Y}$ mice.

Figure S2

A

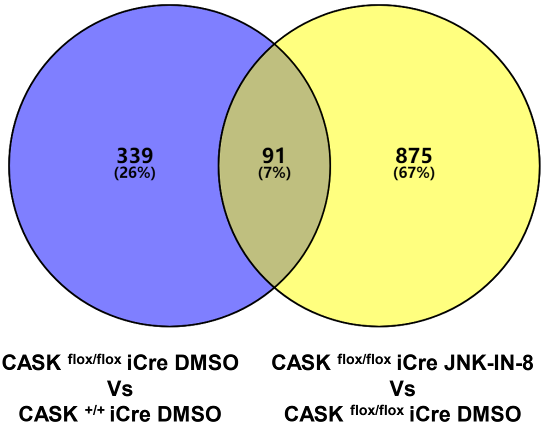

B

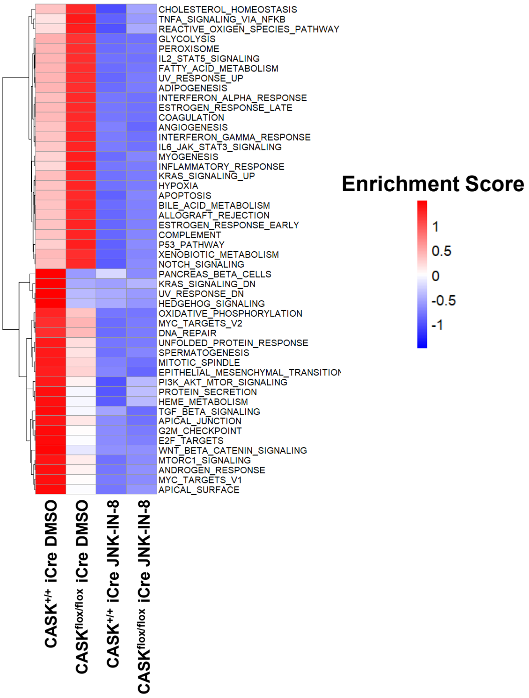

Supplementary Figure S2: Additional results of transcription difference between CASK $^{flox/flox}$ iCre and CASK $^{+/+}$ iCre cerebellum granule (CG) cells

A. Venn map of significantly changed genes in different comparations.

B. Overall gene set enrichment analysis results between four groups, heatmap displays enrichment score changes.

Figure S3

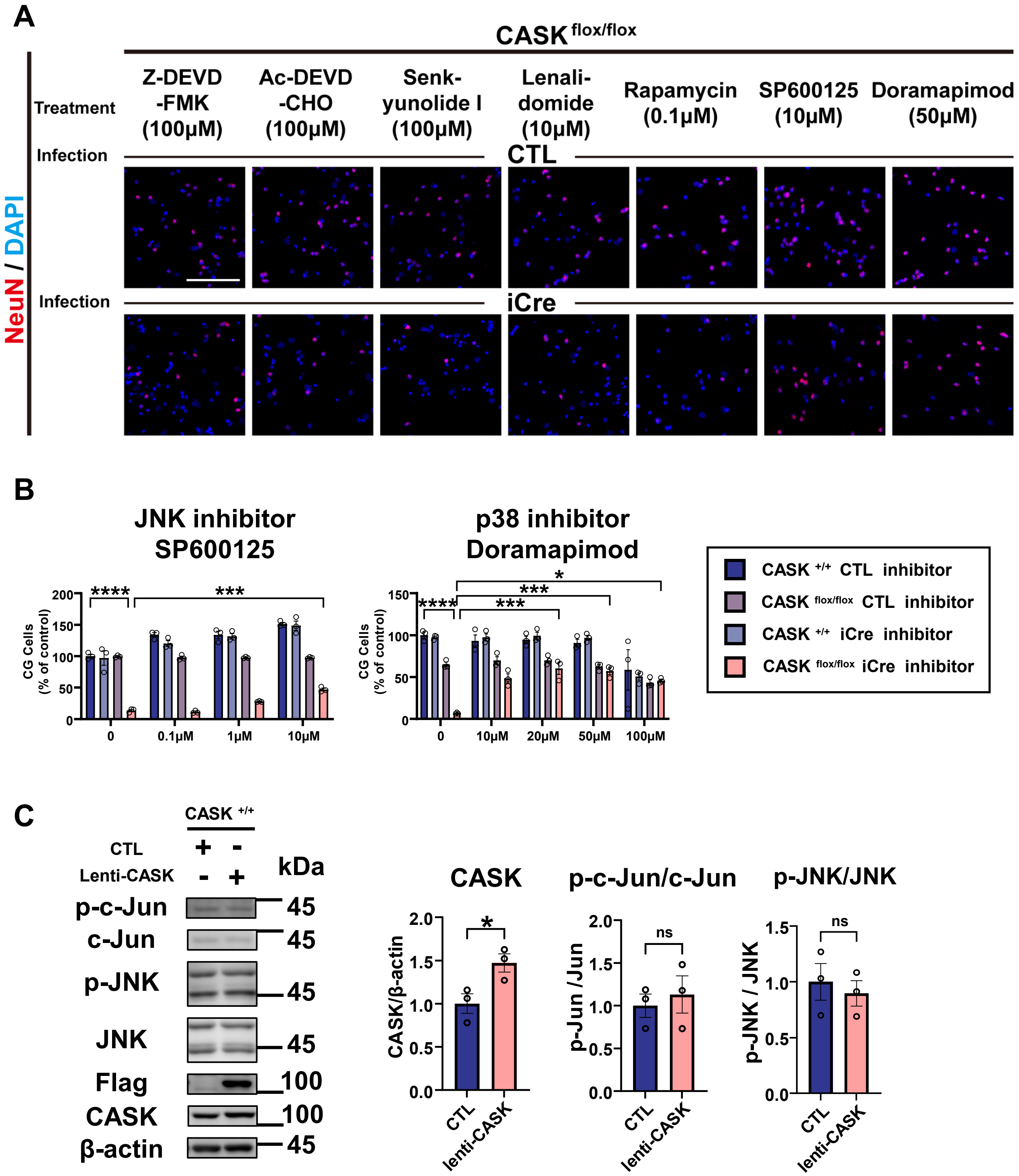

Supplementary Figure S3: JNK, p38 inhibitor may improve CASK$^{flox/flox}$ iCre CG cells survival rates.

A. Additional images of CG cells stained with NeuN and DAPI, scale bar =100 μm.

B. Changes in cell survival rate calculated by the NeuN and DAPI double positive cells. And the group of CASK$^{+/+}$ CTL CG cells with DMSO was used as the control. (n=3)

C. Protein level changes in Lenti-CASK infected CG cells.(n=3)

* indicates difference between the groups. * indicates *p*<0.05; ** indicates *p*<0.01; *** indicates *p*<0.001 and **** indicates *p*<0.0001.

Figure S4

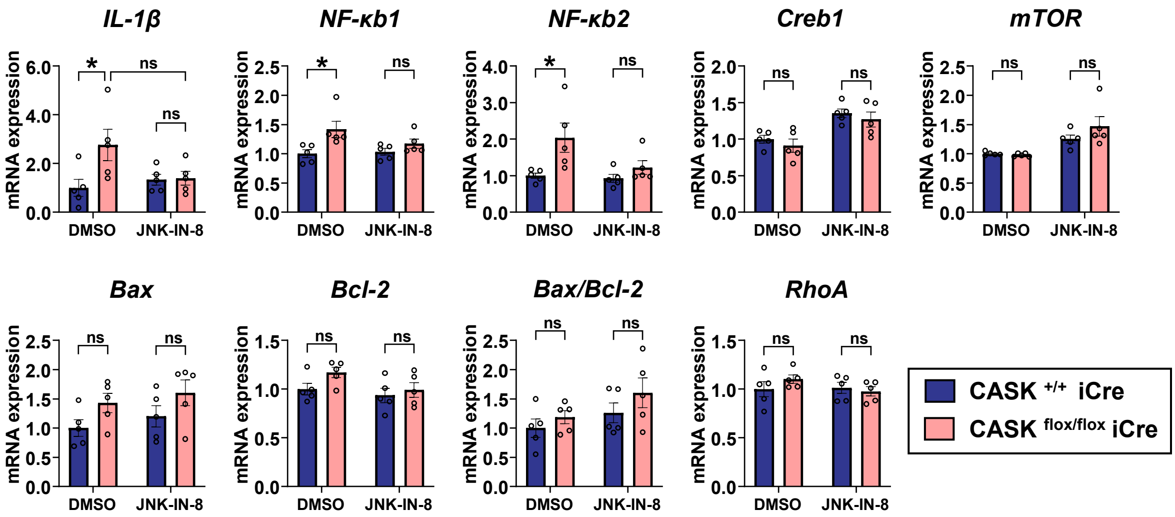

Supplementary Figure S4: Additional results of realtime-qPCR analysis.
CG cells treated with DMSO or JNK-IN-8 was used for realtime-qPCR analysis. (n=5)
* indicates difference between the groups. * indicates p<0.05.

Figure S5

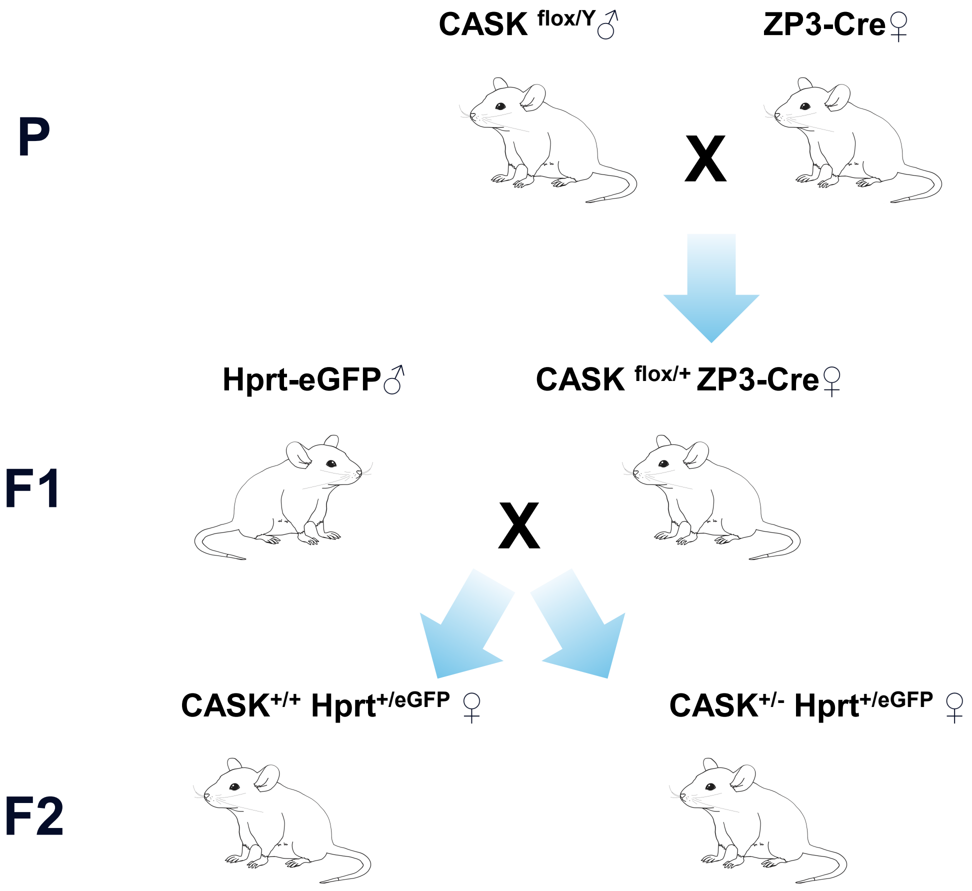

Supplementary Figure S5: Breeding information of heterozygous CASK KO with Hprt-eGFP ($CASK^{+/-}$ $Hprt^{eGFP/+}$) mice

Figure S6

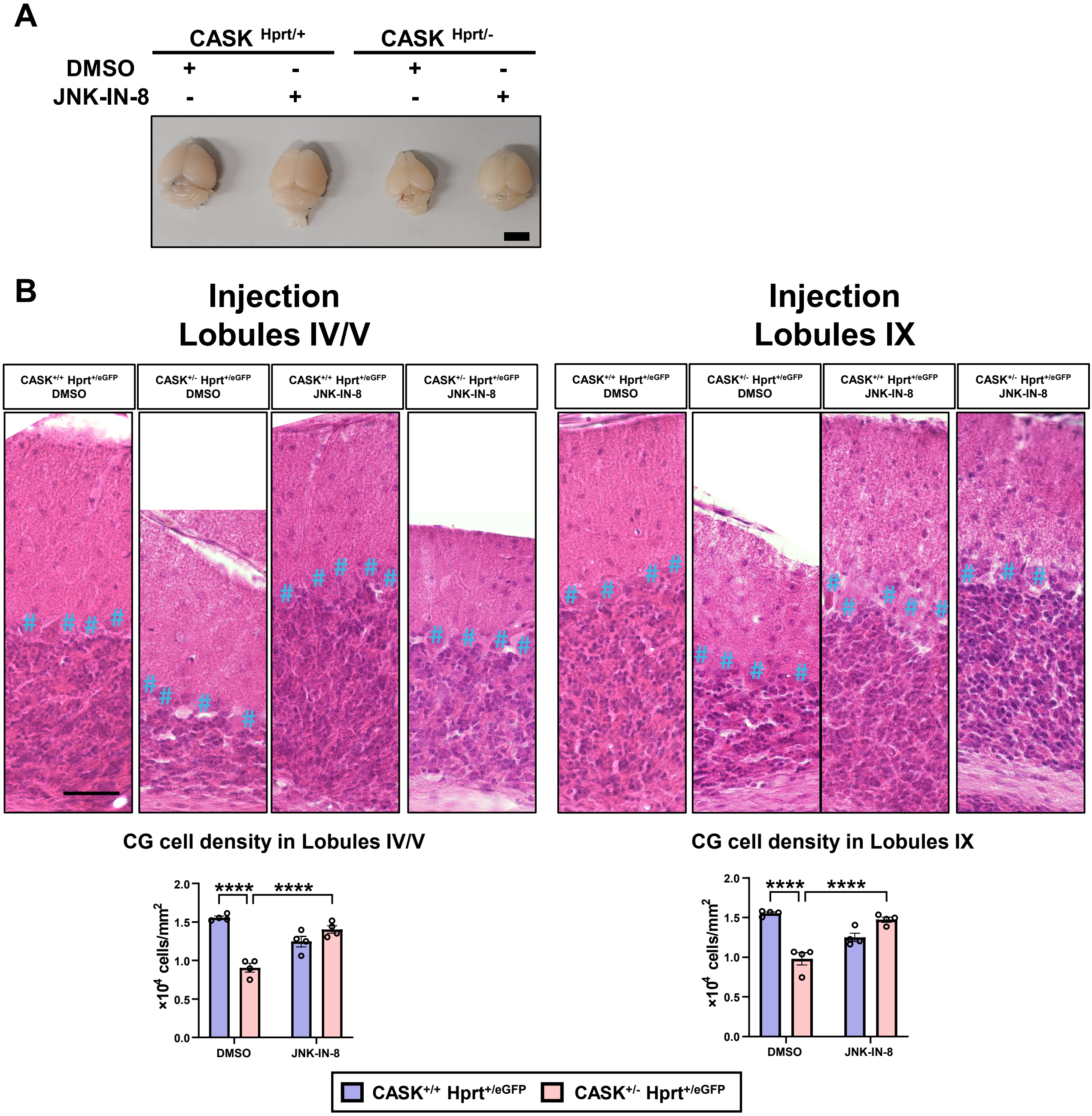

Supplementary Figure S6: Heterozygous CASK KO with Hprt-eGFP (CASK$^{+/-}$ Hprt$^{eGFP/+}$) mice showed cerebellar developmental disorders

A. Photos of cerebellum from mice injected with DMSO or JNK-IN-8, scale bar=1mm.

B. H&E stained Lobules IV/V slices and Lobules IX slices and cerebellum granule (CG) cell density was measured, scale bar=50μm. (n=4) (# indicates the location of Purkinje cells)

* indicates difference between the groups. **** indicates p<0.0001.

Figure S7

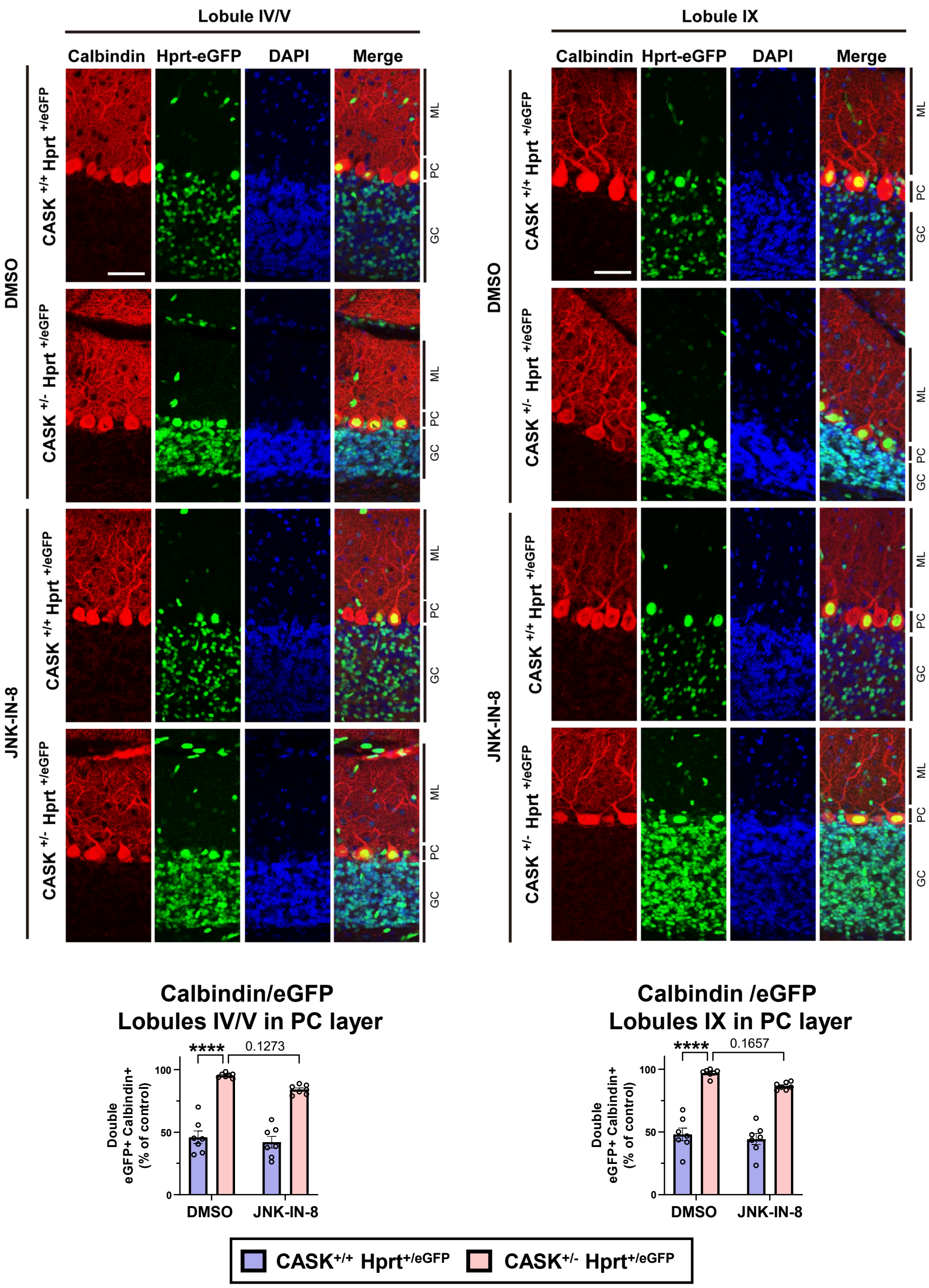

Supplementary Figure S7: JNK-IN-8 reduced the ratio of Calbindin /eGFP double positive cells in Purkinje cell layer minorly.
Immuno*histo*chemistry stained Lobules IV/V slices and Lobules IX. ML, molecular layer; PC, Purkinje cell layer; GC, granular cell layer. The ratio of double positive cells of NeuN and eGFP in PC layer was calculated. (n=7)
* indicates difference between the groups. **** indicates p<0.0001.

Figure S8

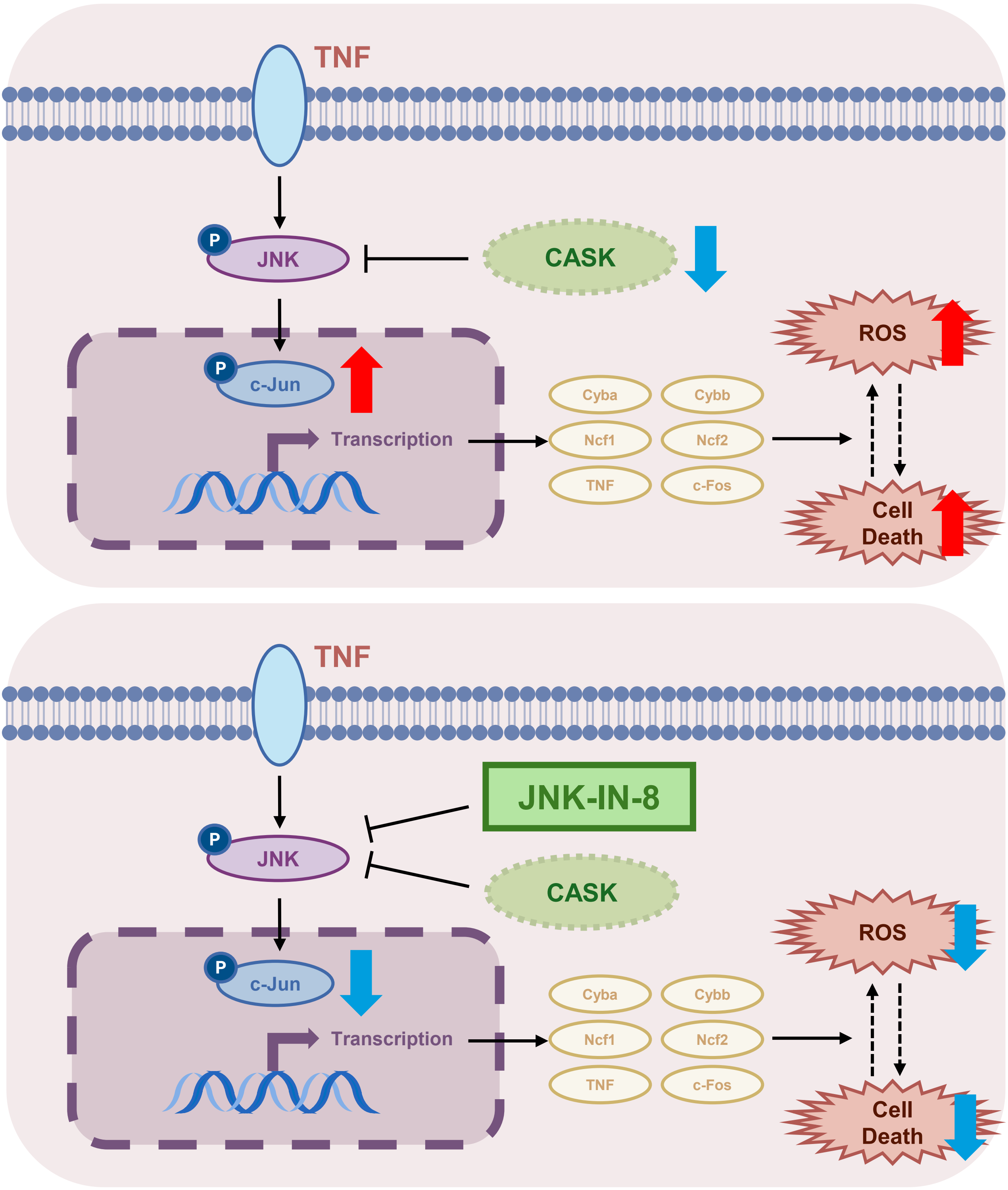

Supplementary Figure S8: Summary of the study.
CASK deletion caused apoptosis and neurodegeneration in CG cells by upregulating JNK signaling, which further promotes apoptosis and ROS related gene transcription. JNK signaling inhibitor suppressed such pathway and increased the CG cells' survival rates.
