# Supplementary material for: Jun N-Terminal Kinase Inhibitor Suppresses CASK Deficiency-Induced Cerebellar Granular Cell Death in MICPCH Syndrome Model Mice
Source: Cells. 2025 May 20;14(10):750. doi: 10.3390/cells14100750 (PMC12109623; doi:10.3390/cells14100750)
Supplement: Supplementary file 1 [file cells-14-00750-s001.zip › Supplementary Methods.pdf]

Supplement methods

Realtime-qPCR

After total RNA was extracted, 1 ug RNA was converted to cDNA using the High-Capacity cDNA Reverse Transcription Kit (ThermoFisher Scientific). 50 ng cDNA was used to perform qPCR using Power SYBR Green PCR Mix (Applied Biosystems, Warrington, UK) on QuantStudio3 (ThermoFisher Scientific). The PCR temperature setting was as follows: 50 °C for 20 min and 95 °C for10min, followed by 40 cycles of 95 °C for 15 s and 60 °C for1min. Themeltcurve stage was 95 °C for 15 s, 60 °C for 1 min, and 95 °C for 15 s. Expression changes were calculated by the delta-delta Ct method, GAPDH were used as an endogenous control. The primer design was listed in table S1.

Western blotting

CG cells were lysis with RIPA buffer (20 mM HEPES pH 7.4, 100 mM NaCl, 1 mM EDTA, 1% Triton-X 100 containing the protease inhibitor cocktail (Nacalaitesque, Kyoto, Japan). The protein concentration of the CG cell lysate was determined by the BioRad Protein Assay System (BioRad, Berkeley, CA, USA). After adjusting, 20 μg protein was subjected to SDS-PAGE (7.5% Laemmli) and electroblotted onto Immobilon-FL PVDF membrane (Millipore, Burlington, MA, USA). The membrane was incubated with the first antibody at 4°C, overnight. On the next day, the second antibody was added and incubated with the membrane at 25°C 1h. The antibodies used in the study are listed in table S2. The Phosphorylation protein solution used the Can get signal (NKB-101, TOYOBO, Japan). Visualized by ODYSSEY Imaging System (LI-CDR Bioscience, Lincoln, NE, USA). Quantification by the ImageJ (version 2.3.0, FIJI, NIH, USA).

Reactive Oxygen Species Detection

To detect Reactive Oxygen Species in CG cells at DIV5, ROS Assay Kit -Highly Sensitive DCFH-DA- (R252, DOJINDO LABORATORIES, Kumamoto, Japan) was employed, according to the manufacturer's instructions. CG cells were seeded at a density of 2×10^5 cells/well in a 24-well plate (83.3922, SARSTEDT AG & Co. KG, Nümbrecht, Germany). After DIV5, cells were pre-treated with Highly Sensitive DCFH-DA Dye for 30 min at 37℃, 5 % CO_2 incubator. After washed three times with the HBSS-Hanks'

Balanced Salts Solution (HBSS), images were acquired with a Keyence microscope (BZ-X810, Keyence, Osaka, Japan) and quantification was conducted by using ImageJ.

Hematoxylin & Eosin Staining

P0 $CASK^{+/Y}$, $CASK^{-/Y}$ and P6 $CASK^{+/flox}Hprt^{eGFP/+}$, or $CASK^{+/+}Hprt^{eGFP/+}$ mice were transcranial perfused with 4% PFA in PBS. After brains were removed, postfixed in 4% PFA, 24 h later incubated in the 15% sucrose in PBS, 24 h later incubated in the 30% sucrose, embedded in Tissue Tek compound, and stored at −80 ˚C. Successive 20 µm thick sagittal sections were prepared using a cryostat microtome (Leica Microsystems, Wetzlar, Germany, CM1950), and stained with hematoxylin and eosin (H&E). Images were acquired with a Keyence microscope (Keyence BZ-X810). Images of the whole cerebellum were taken using a 40× lens. The CG cell density (cells/area) in cerebellum lobules IV/V and IX area were analyzed using ImageJ.

NeuN and Calbindin Immuno*cyto*chemistry and Immuno*histo*chemistry

The cultured cells were fixed with 4% PFA/ 4% sucrose in PBS at DIV7, and immunostained with a Mouse Anti-NeuN antibody (Sigma-Aldrich, MAB377, 1:1000 cell culture, 1:500 brain slice), Anti-Calbindin-D28k antibody (1:200) followed by incubation with Donkey anti-Mouse IgG H&L (Alexa Fluor® 594) (Abcam, Cambridge, UK, ab150108, 1:1000). Fluorescence images were acquired with a confocal laser scanning microscope (TCS SP8; Leica Microsystems). CG cells were identified based on NeuN signals and their morphological features, small size (5–10 µm in diameter), regular round or ovoid shape. mouse anti-tubulin3/Tuji1 (Genetex, Irvine, CA, USA, 1:1000) antibodies, followed by incubation with Donkey anti-rabbit IgG H&L (Alexa Fluor® 594) (Abcam, 1:500) and Donkey anti-Mouse IgG H&L (Alexa Fluor® 647) (Abcam, 1:500).

TUNEL Assay

Terminal deoxynucleotidyl transferase-mediated dUTP nick end labeling (TUNEL) assay was performed using the in situ Cell Death Detection kit, Fluorescein (Roche, Basel, Switzerland), according to as described previously[1]. The CG cells were fixed at DIV7 and incubated in 0.25% Triton X-100 (Nacalai tesque) at 25˚C for 10 min, followed by incubation with reaction solution and DAPI at 37˚C for 60 min. Fluorescence images were acquired with a confocal laser scanning microscope (TCS SP8; Leica Microsystems), quantified using ImageJ.

1. Guo Q, Kouyama-Suzuki E, Shirai Y, Cao X, Yanagawa T, Mori T, Tabuchi K. Structural Analysis Implicates CASK-Liprin-alpha2 Interaction in Cerebellar Granular Cell Death in MICPCH Syndrome. *Cells* 2023, **12**(8).
